# SAKE-PP: A Spatial-Attention Equivariant Network for Accurate Ranking of Protein–Protein Interaction Models

**DOI:** 10.1021/jacsau.6c00166

**Published:** 2026-04-16

**Authors:** Yuzhi Xu, Wei Xia, Chao Zhang, Xinxin Liu, Cheng-Wei Ju, Xuhang Dai, Pujun Xie, Yuanqing Wang, Guangyong Chen, John Z.H. Zhang

**Affiliations:** † NYU-ECNU Center for Computational Chemistry, 5894 NYU Shanghai, Shanghai 200126, China; ‡ Department of Chemistry, New York University, New York, New York 10003, United States; § Faculty of Synthetic Biology, 704566 Shenzhen University of Advanced Technology, Shenzhen 518107, China; ∥ Hangzhou Institute of Medicine, 631027 Chinese Academy of Sciences, 150 Dongfang Street, Xiasha, Qiantang District, Hangzhou, Zhejiang 310018, China; ⊥ Pritzker School of Molecular Engineering, 2462 The University of Chicago, Chicago, Illinois 60615, United States; # Department of Biochemistry and Molecular Pharmacology, 214373 New York University Grossman School of Medicine, New York, New York 10016, United States; ∇ Collaborative Innovation Center of Extreme Optics, Shanxi University, Taiyuan 030006, China

**Keywords:** Deep Learning, Protein−Protein Complexes, Molecular Dynamics, iRMSD Prediction, Scoring Function

## Abstract

Accurate prioritization of near-native protein–protein interaction (PPI) models remains a major bottleneck in structural biology. Here, we present SAKE-PP, a physics-inspired, spatial-attention equivariant graph neural network that directly regresses interface RMSD (iRMSD) without native references. Trained with a hierarchical iRMSD-guided sampling strategy on PDBBind, SAKE-PP integrates force-field-like attention with Laplacian-eigenvector orientation to couple local interaction forces with global topology. On the 2024PDB benchmark of 176 heterodimers, SAKE-PP improves AF3-decoy selection by 13.75% (iRMSD) and 12.5% (DockQ) and consistently outperforms the AF3 ranking score in overlap, hit-rate, and correlation metrics. In zero-shot evaluation on 139 antibody–antigen complexes, SAKE-PP increases correlation by 0.4. By promoting geometrically near-native, energetically plausible interfaces to the top ranks, SAKE-PP reduces wasted MD trajectories and improves refinement reliability. Overall, SAKE-PP provides a robust, plug-and-play scoring function that streamlines PPI evaluation and accelerates downstream structure-guided drug-design workflows.

## Graphical abstract

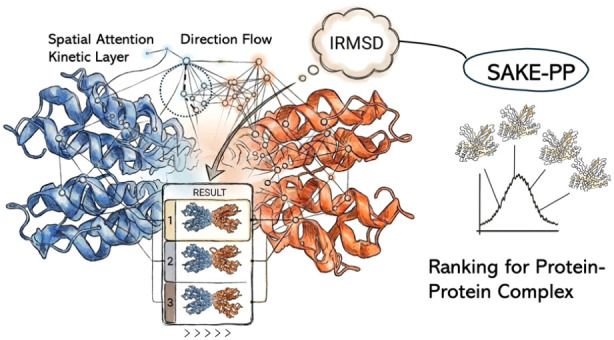

## 1 Introduction

Protein–protein interactions (PPIs) underpin essential cellular processes, and resolving their structural and energetic determinants is central to mechanistic biology and therapeutic discovery.^1–4^ Although crystallography and cryo-EM provide atomic-level insights, their applicability is often limited by protein flexibility, heterogeneity, and assembly size.^5,6^ Consequently, computational tools—including docking and molecular dynamics (MD)—have become indispensable for probing PPI structure and dynamics.^7,8^ A key evaluation metric for these computational predictions is the interface RMSD (iRMSD), which quantifies interface accuracy and also serves as a crucial readout of MD stability.^9,10^ However, robustly achieving low iRMSD across diverse complexes remains challenging, spotlighting the need for more accurate and efficient scoring strategies.^11,12^

Deep-learning predictors, most notably AlphaFold and AlphaFold-Multimer, have reshaped protein structure modeling.^13,14^ AlphaFold3 (AF3) further generalizes to biomolecular assemblies, enabling large-scale generation of candidate models for protein–protein, protein–ligand, and protein–nucleic-acid complexes.^15^ For complexes, AF3 reports confidence signals such as pTM/ipTM and uses them to rank multiple sampled structures. However, these internal scores are learned as model-confidence indicators rather than as direct surrogates of docking-quality metrics and can therefore be misaligned with interface accuracy. This motivates a key question: can we predict a rigorous interface-quality measure, most notably interface RMSD (iRMSD), in a reference-free manner to reliably select near-native complexes from large decoy ensembles?

Accurate iRMSD estimation would be especially valuable as a practical gatekeeper for downstream refinement, because it prioritizes geometrically correct interfaces that are more likely to remain stable under molecular-dynamics (MD) simulations and yield meaningful energetics. While reference-free error regression has been explored for protein–ligand complexes or single-chain proteins (e.g., DeepBSP, RmsdXNA, DeepAccNet),^16–18^ extending it to PPIs remains difficult: docking errors arise from subtle relative reorientations of two large bodies, and iRMSD is an unbounded physical distance with heavy-tailed decoy distributions.^19^ Consequently, the field often favors bounded composite metrics such as DockQ for evaluation, yet a continuous iRMSD predictor would provide a more actionable signal for selecting near-native starting structures and improving ensemble-based PPI modeling.

In this paper, we introduce SAKE-PP, a reference-free iRMSD predictor tailored for protein–protein interactions, enabling Ångstrom-level scoring of large decoy sets and seamless integration with physics-based refinement. SAKE-PP is an independent, physics-inspired spatial-attention equivariant GNN that avoids biases from structure-prediction frameworks such as AF3. Its architecture combines a Spatial Attention Kinetics module, which jointly updates node features and 3D coordinates using force-field–like attention, with a Laplacian eigenvector Orientation module that encodes global fold topology; multiscale fusion yields a unified interface representation.

To construct a robust training set, we generated 1.47M docking poses for 736 PDBBind complexes and applied hierarchical sampling to obtain 15,456 diverse, near-native conformations. SAKE-PP consistently surpasses state-of-the-art GNN variants and strongly improves AF3 decoy selection on the 2024PDB benchmark, achieving 13.75% gains in iRMSD statistics and 12.5% in DockQ. It outperforms AF3 scores across overlap, hit rate, and correlation metrics and generalizes well to 139 unseen antibody–antigen complexes, improving score–iRMSD correlation by 0.4. As iRMSD correlates with MM/GBSA and FEP energetic trends, SAKE-PP provides a physically meaningful, continuous signal for selecting stable, high-affinity conformers. Altogether, SAKE-PP delivers a plug-and-play scoring function that enhances PPI evaluation and accelerates downstream structure-guided drug discovery (Figure 1).

**Figure 1:**
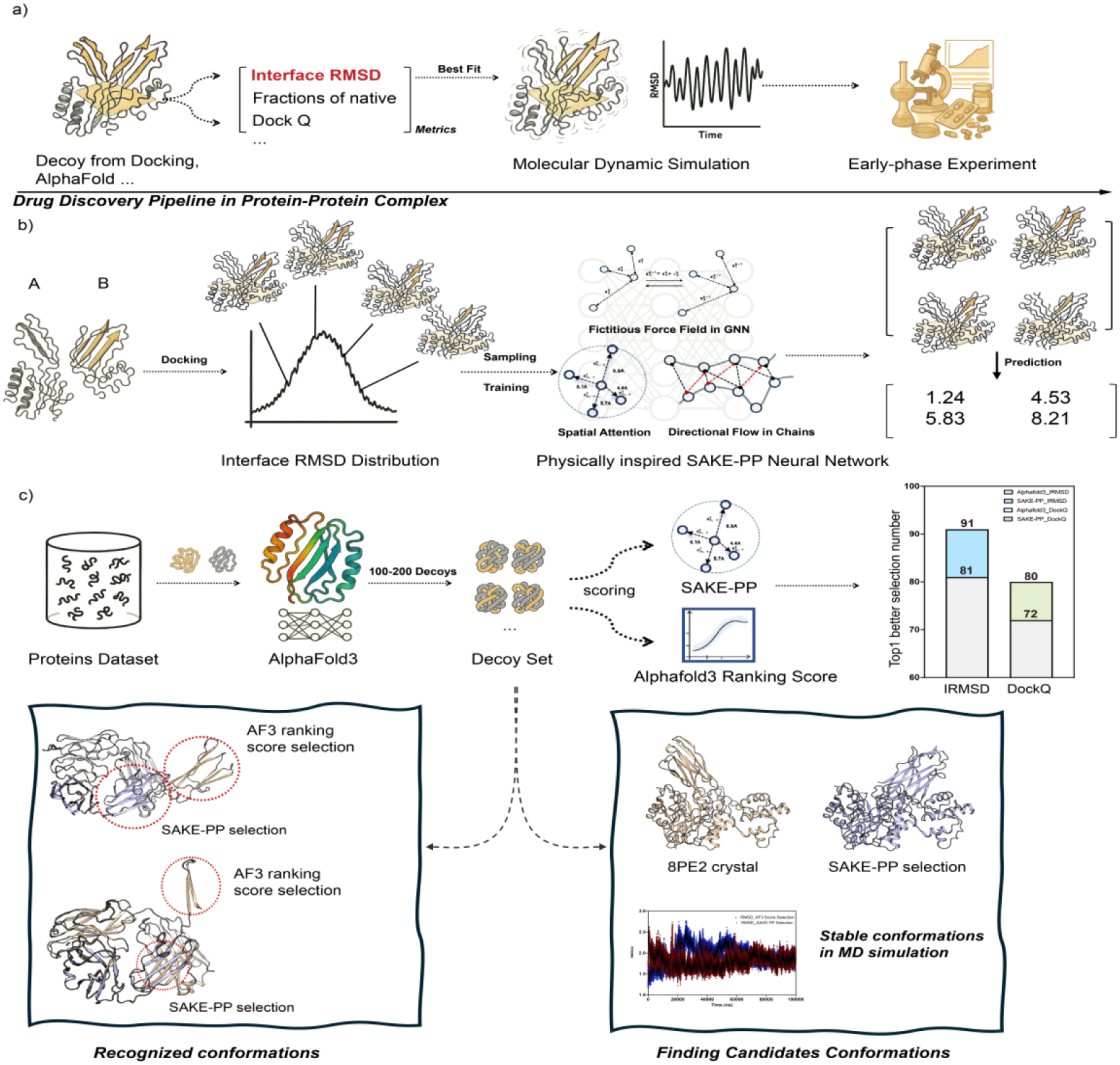
(a) In a typical protein–protein complex drug discovery pipeline, candidate structures are first generated via docking or AI-based prediction methods such as AF3. Each decoy is then evaluated using metrics such as interface RMSD (iRMSD), fraction of native contacts, or the composite DockQ score. Top-scoring conformations are subsequently subjected to all-atom molecular dynamics (MD) simulations and early phase experimental validation. (b) SAKE-PP training workflow: Given a set of docking-generated complexes between proteins A and B, an iRMSD distribution is obtained, from which representative decoys are selected via hierarchical sampling strategies to train SAKE-PP. SAKE-PP is a physics-inspired, geometrically equivariant graph neural network that learns to predict iRMSD values for unseen decoys, guiding the identification of near-native binding modes. (c) Cross-data set benchmarking and representative examples: For a diverse set of protein targets, AF3 is used to generate 100 decoys per target, which are then independently scored by SAKE-PP and AF3 ranking score. The bar chart shows the number of targets for which SAKE-PP (colored bars) or AF3 ranking score (gray bars) selects the top 1 decoy under iRMSD or DockQ. Bottom left: Comparison of SAKE-PP-selected low-energy conformations versus AF3 ranking score-selected high-energy, unstable alternatives for the same target. Bottom right: MD simulations of PDB 8PE2 over 1 μs show that structurally distinct conformations selected by different scoring functions remain stable, suggesting the existence of multiple coexisting low-energy states in the protein–protein interaction landscape.

## 2 Methods

### 2.1 Data Preparation and Preprocessing

Two-chain protein–protein complexes were extracted from the PDBBind data set (PDBBindv2020).^20,21^ Structures were cleaned with pdb4fixer (removing water and hetero atoms, retaining protein atoms only). For length-imbalanced pairs, the shorter chain was defined as the ligand and the longer as the receptor, yielding 736 complexes. Each complex was docked by ZDock (2000 poses per complex).^22^ To restrict the search to a plausible interface, we imposed a 15 Å ligand–receptor distance restraint defined by the closest native interchain contact, producing 1,472,000 poses in total.

To mitigate the strongly skewed pose-quality distribution and enrich the near-native regime (CAPRI iRMSD <4 Å; typical modes around 4–5 Å),^23^ we computed iRMSD, fnat, and DockQ for all poses and performed iRMSD-centered hierarchical sampling. If sufficient poses existed within 0–5 Å, we sampled the 0–5 Å range by 10% quantiles and added uniform samples from 5–8 Å; otherwise, we kept the top 10 poses within 0–8 Å and uniformly filled to 20 (or retained all if fewer than 20). Together with native structures, this yielded 15,456 conformations.

After sampling, 42.87% of poses are acceptable (iRMSD <4 Å) and 57.13% unacceptable by CAPRI criteria; within the acceptable set, 34.01% fall in 0–2 Å and 65.99% in 2–4 Å (Figure 2b–d).

**Figure 2:**
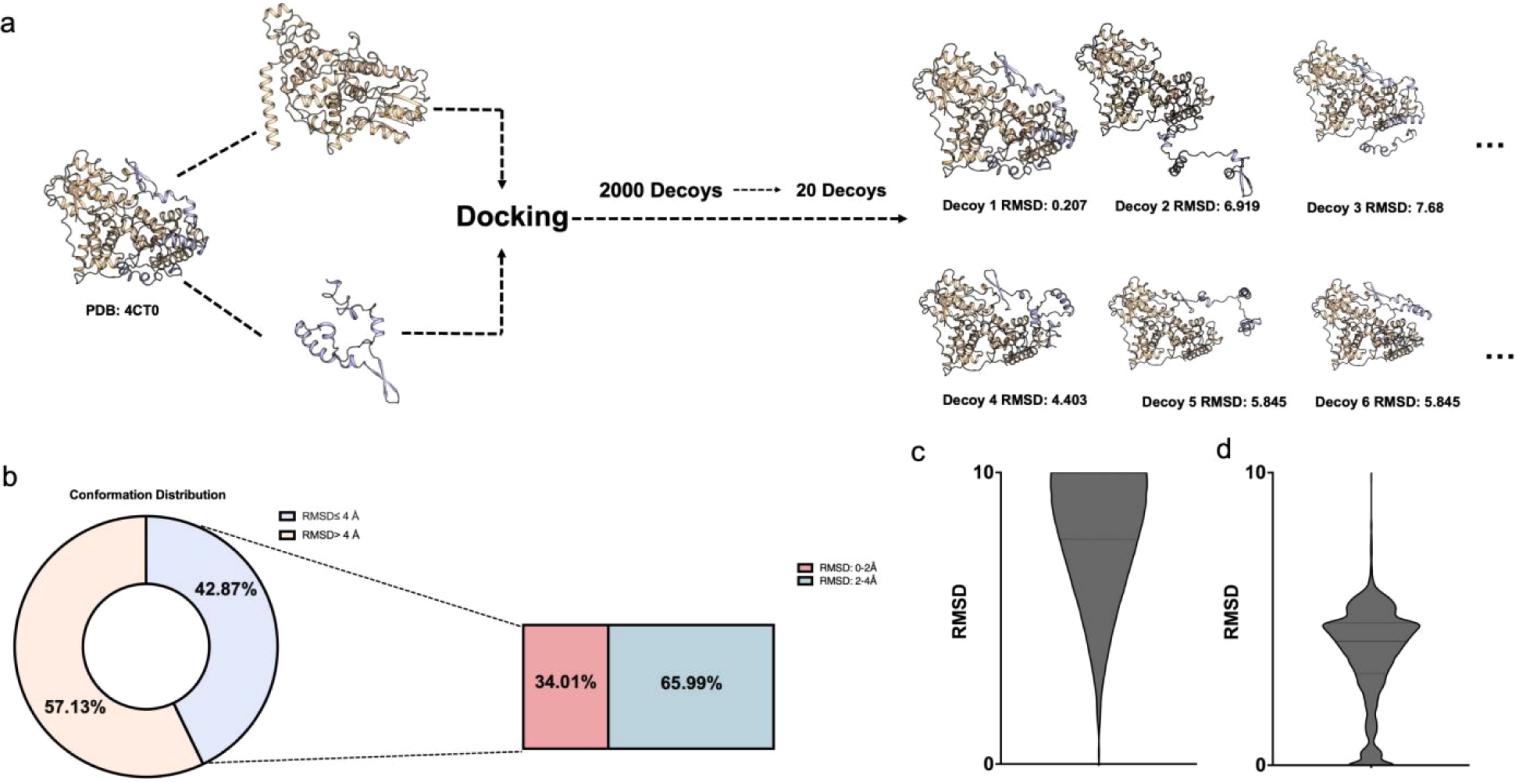
(a) From PDBBind, 736 protein–protein complexes were docked using ZDock with a 15 Å distance restraint, generating 2,000 decoys per complex, followed by hierarchical sampling to retain 20 representative poses. Right: representative decoys for PDB 4CT0 with their iRMSD values. (b) Conformational distribution after sampling. By CAPRI criteria (iRMSD ≤4 Å vs >4 Å), 42.87% of decoys are acceptable (light blue) and 57.13% are unacceptable (peach). Within the acceptable set, high-quality poses (0–2 Å; 34.01%, red) and borderline poses (2–4 Å; 65.99%, blue) are shown. (c) iRMSD distribution of all 1,472,000 decoys within 0–10 Å, exhibiting a “sandglass”-shaped distribution with enrichment at high RMSD. (d) iRMSD distribution after hierarchical sampling, showing more balanced coverage and enrichment in the critical 0–4 Å range.

### 2.2 Workflow of SAKE-PP

SAKE-PP adopts a dual-stream architecture to score protein–protein docking quality by regressing iRMSD from an interface-centered residue graph representation of the complex. Starting from the interface neighborhood, each residue node is encoded with amino-acid identity, backbone dihedrals (ϕ,ψ), Cα coordinates, and distance-derived geometric descriptors, while edges capture local inter-residue geometry through relative displacements and radial distance encodings. These features are processed in parallel by two complementary branches (Figure 3).

1 The Spatial Attention Kinetic (SAKE) stream performs geometry-aware attention over neighboring residues, where semantic interactions are modulated by edge-conditioned weights and radial encodings, and jointly refines representation and geometry via a physics-inspired kinetic update: each residue maintains a learned velocity that is iteratively evolved under a fictitious force field defined as a weighted aggregation of equivariant pairwise displacement vectors, enabling coupled updates of node embeddings and coordinates while preserving *E*(*n*)-equivariance.
2 In parallel, the Direction Flow stream models chain-aware, multidirectional information propagation by incorporating directional/angle relations to facilitate structured message passing across chains, thereby capturing long-range interchain dependencies beyond purely distance-based aggregation.

**Figure 3:**
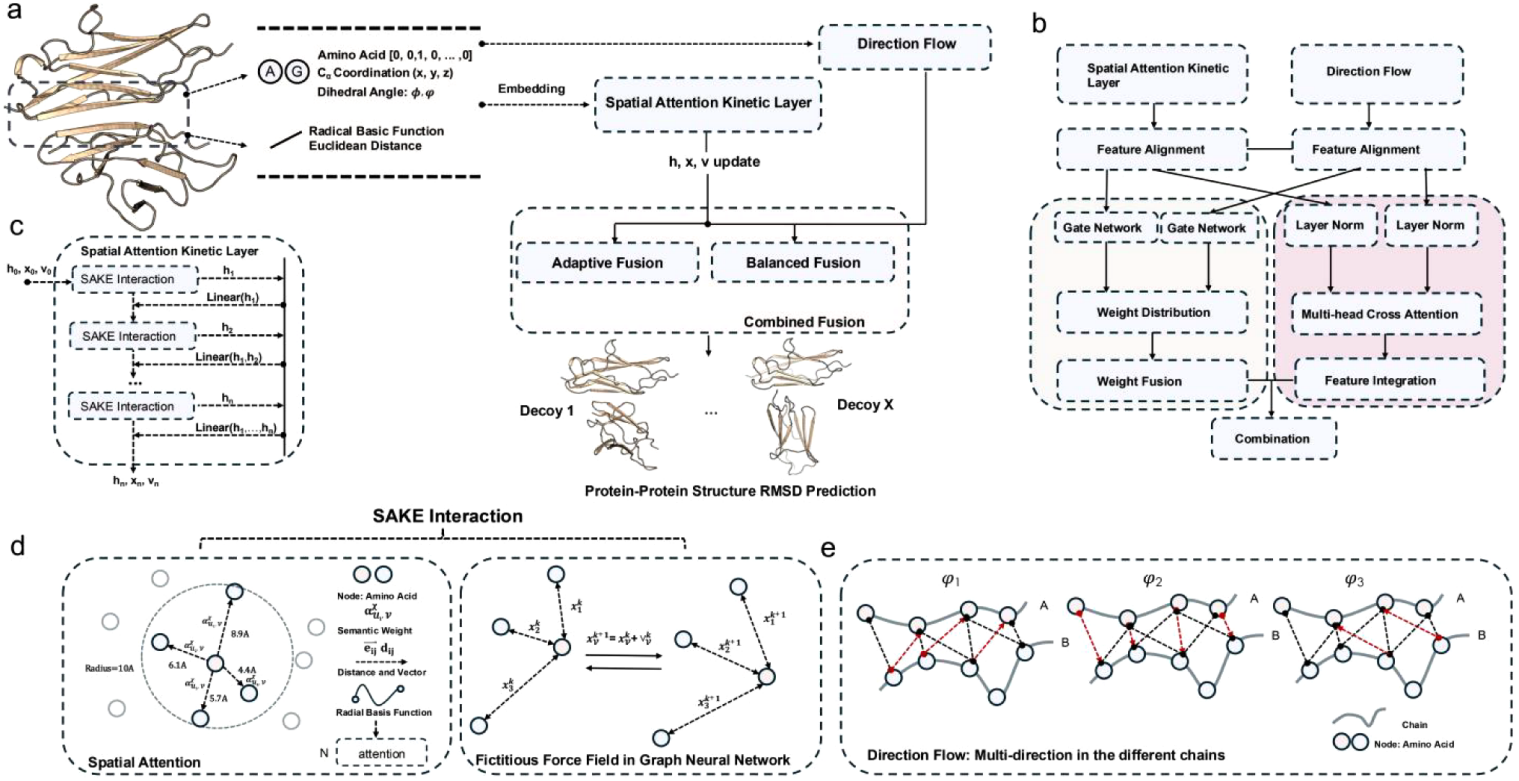
Workflow and architecture of SAKE-PP for protein–protein structure iRMSD prediction. (a) Workflow of SAKE-PP: Each protein complex is encoded using amino acid type, dihedral angles (ϕ,ψ), Cα coordinates (*x*, *y*, *z*), and Euclidean distance–based features within a 10 Å interface region. The representations are passed to both the Direction Flow and the Spatial Attention Kinetic Layer. Their outputs are integrated in the Combined Fusion module to predict a scalar iRMSD value. (b) Feature fusion mechanism: The two representation streams undergo feature alignment and are processed by gate networks, weighted fusion, and multihead cross attention to yield a unified latent representation. (c) Spatial Attention Kinetic Layer: Composed of stacked SAKE Interactions, each connected via residual links. Each SAKE layer updates node features **h**, positions **x**, and velocities **v** in an *E*(*n*)-equivariant manner. (d) SAKE Interaction Module: Integrates spatial attention and a fictitious force field for joint node and coordinate updates. The spatial attention mechanism combines radial basis encoding of pairwise distances with edge-based semantic attention, capturing local geometric dependencies. A mixed attention score $\alpha_{uv}^{X\times H}$ modulates neighborhood aggregation. Position updates are coupled with learned velocity vectors that evolve under a physics-inspired force field: $x_{v}^{\left(k+1\right)}=x_{v}^{\left(k\right)}+v_{v}^{\left(k\right)}$ , $v_{v}^{\left(k+1\right)}=\phi^{v\to V}\left(h_{v}^{\left(k\right)}\right)v_{v}^{\left(k\right)}+\underset{u\in N\left(v\right)}{\sum }\lambda_{i}\left(h_{e_{uv}}^{\left(k\right)}\right)f\left({\mathrm{e⃗}}_{uv}^{k}\right)$ . (e) Direction Flow: Captures multidirectional spatial interactions across protein chains using chain-aware angular relationships (φ_1_, φ_2_, φ_3_) to guide geometric feature propagation.

The two streams are then aligned and fused through gated and weighted integration augmented with cross-attention to form a unified latent representation, from which the model regresses a single iRMSD value as the docking-quality metric; further formulation details are provided in of Section 3 of the Supporting Information.

## 3 Results and Discussion

### 3.1 Comparison of Baseline Methods

Table 1 compares SAKE-PP with representative GNN baselines, where all variants share the same input representation set mentioned in Section 3 of the Supporting Information, training protocol and evaluation pipeline, differing only in the choice of graph neural network backbone, under 5-fold and 10-fold cross-validation. SAKE-PP delivers the best overall performance with strong split-wise stability (R in 5-fold: MAE 0.9451 ± 0.0157, 0.6164 ± 0.0118; R in 10-fold: MAE 0.9466 ± 0.0336, R 0.6240 ± 0.0206), outperforming EGNN/DGN and conventional GNNs (GCN/GAT/HGT). Its consistently smaller standard deviations indicate reduced sensitivity to data partitioning and more reliable ranking.

**Table 1:** Model Comparison Under 5-Fold and 10-Fold Cross-Validation^a^

5-Fold	MAE	RMSE	R
Our Model	**0.9451 ± 0.0157**	**1.2912 ± 0.0275**	**0.6164 ± 0.0118**
EGNN^24^	0.9922 ± 0.0203	1.2986 ± 0.0555	0.5983 ± 0.0071
DGN^25^	0.9966 ± 0.0796	1.2886 ± 0.2507	0.6035 ± 0.0810
GCN^26^	1.0983 ± 0.0186	1.423 ± 0.0490	0.4933 ± 0.0129
GAT^27^	2.0448 ± 0.2599	1.9126 ± 0.3420	0.0021 ± 0.0177
HGT^28^	1.2081 ± 0.0179	1.575 ± 0.0066	0.0059 ± 0.0240

**10-Fold**	**MAE**	**RMSE**	**R**
Our Model	**0.9466 ± 0.0336**	**1.2639 ± 0.0357**	**0.6240 ± 0.0206**
EGNN	0.9892 ± 0.0292	1.2705 ± 0.1119	0.6034 ± 0.0244
DGN	0.9860 ± 0.0932	1.2787 ± 0.3093	0.6094 ± 0.0831

a Results are reported as mean ± Std. Bold indicates the best performance, and underlining indicates the second-best.

### 3.2 Comparative Analysis of SAKE and AF3 Ranking Score in Protein–Protein Complex Structure Selection

AlphaFold 3 evaluates complex reliability using pTM/ipTM scores (0–1) and ranks multiple samples with a composite score (ranking score = 0.8 × ipTM + 0.2 × pTM + 0.5 × disorder – 100 × clash). Although SAKE-PP was trained on rigid-body ZDOCK decoys, we hypothesize that it transfers to flexible AlphaFold 3 decoy discrimination by leveraging interface-geometry cues. We curated 176 binary complexes from 2024 PDB releases (with post-June entries emphasized) using RCSB FASTA identifiers, with basic cleaning and standardization (removing chains <20 residues, PDB4Fixer cleanup, and mapping noncanonical residues). For each complex, we generated 100 AlphaFold 3 decoys across 20 seeds (17,600 total), computed within-complex iRMSD, and constructed a homology-stratified test set (20% homologous vs 80% heterologous receptors.

#### 3.2.1 Overall Analysis

Figure 4 compares SAKE-PP with the AlphaFold 3 (AF3) ranking score for prioritizing AF3-generated decoys. For Top-1 selection, SAKE-PP chooses a better decoy by iRMSD in 91/176 cases versus 81/176 for AF3 (4 ties; + 13.75%), and by DockQ in 80/176 versus 72/176 (+12.5%), indicating stronger enrichment of high-quality structures. Beyond Top-1, we assessed the agreement between the two ranking schemes using the Top-k overlap rate (Figure 4c,d), defined as the fraction of targets for which the Top-*k* lists (SAKE-PP or AF3), when compared against the ground-truth ranking, share at least one common decoy; this reflects how consistently each method recovers MD-relevant candidates. SAKE-PP dominates AF3 across all *k*, with a 67% relative gain at *k* = 1, remaining superior through *k* = 5, and showing larger margins for *k* ≥ 6 (absolute gain >5 pp; relative peak 18% at *k* = 6 and ≥14% through *k* = 12), consistent with recovering high-quality decoys missed by AF3. At *k* = 15, SAKE-PP reaches 86.93% overlap versus 77.84% for AF3 (+9.09 pp), and the curves never intersect, supporting globally superior ordering for ensemble free-energy/MD workflows.

**Figure 4:**
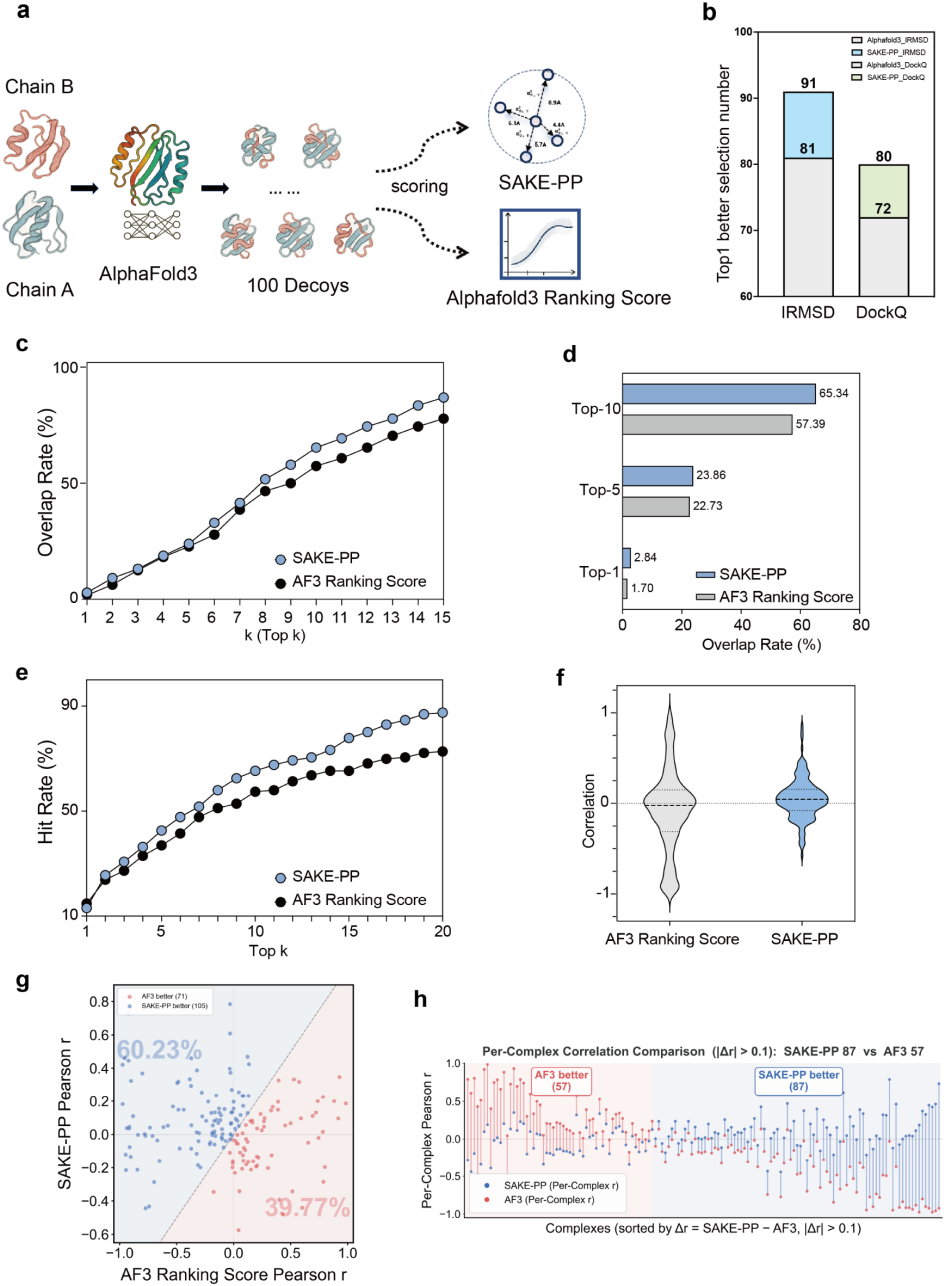
Benchmarking SAKE-PP against the AF3 ranking score on a 176-complex docking set. (a) Workflow schematic. For each heterodimeric target, AF3 generates 100 decoys that are rescored independently by SAKE-PP and AF3 ranking score. (b) Top-1 selection quality. Bars indicate the number of complexes for which the top-ranked decoy chosen by a given method exhibits the lower iRMSD (left) or the higher DockQ score (right). (c) Overlap rate summarized at Top-1, Top-5, and Top-10. (e) Success-hit-rate curves: probability of finding at least one of the ten lowest-iRMSD decoys within the top-k ranks (1 ≤ *k* ≤ 20). (f) Distribution of Pearson correlations (*r*) between predicted score and iRMSD for each complex. (g) Per-complex Pearson *r* comparison. Each point is one complex. SAKE-PP outperforms AF3 ranking score in 105/176 cases (60.23%). (h) Sorted per-complex Δ*r* between SAKE-PP and AF3 ranking score. For |Δ*r*| > 0.10, SAKE-PP wins 87 cases versus 57 for AF3.

Following CAPRI, we define the 10 lowest-iRMSD decoys per target as reference;^29^ *success-hit-rate* is the probability of retrieving at least one reference within Top-k (Figure 4e). SAKE-PP exceeds AF3 from *k* ≥ 2, with mean success-hit of 29.7% vs 27.2% for *k* = 1–5 (+9.2% relative), 71.1% vs 61.2% for *k* = 6–20 (+16.2%), and 60.7% vs 52.7% over *k* = 1–20 (+8.0 pp; + 15.3%); at *k* = 20 the lift is 14.8 pp (87.5% vs 72.7%, + 20%). This improvement is robust (paired t = 7.83, *p* ≈ 2.3 × 10^–7^; Cohen’s *d* = 1.75).

To quantify global score–quality tracking, we computed per-target Pearson *r* between the raw score and iRMSD: SAKE-PP yields higher *r* in 105/176 cases (60.23%) versus 71/176 (39.77%) for AF3, a 51.4% relative improvement in the win–loss ratio (Figure 4g). With |Δ*r*| > 0.10 (advanced threshold), SAKE-PP still wins in 87 complexes versus 57 losses (Figure 4h), and a two-sided Wilcoxon signed-rank test confirms significance (*Z* = 7.35, *p* ≈ 1.9 × 10^–13^). Collectively, SAKE-PP provides stronger early enrichment and more robust correspondence with structural accuracy, improving identification of near-native complexes in high-throughput modeling workflows.

#### 3.2.2 Successful Case

To illustrate the practical utility of SAKE-PP, we analyzed three challenging complexes (8S4K,^30^ 8SOZ,^31^ and 8VGG^32^). The top-1 models selected by the AF3 ranking score exhibit large errors (iRMSD = 17.886 Å, 16.352 Å, and 4.719 Å, respectively; all above the acceptable threshold of 4.0 Å). In contrast, SAKE-PP identifies near-native decoys from the same AF3 ensembles with iRMSD = 1.521 Å, 1.885 Å, and 0.925 Å, respectively.

We also found that this discrepancy is not due to a lack of accurate AF3 decoys but to ranking bias. Specifically for 8S4K, the AF3 ensemble shows a bimodal iRMSD distribution, with 87% clustered at 17.26 Å (“deviated”) and 13% at 1.526 Å (“reasonable”), yielding a mean of 15.22 Å. AF3 selects from the deviated mode (ranking score 0.8709), whereas SAKE-PP selects from the reasonable mode (AF3 score 0.8385; Figure 5a), differing mainly by a displacement at a key structural region.

**Figure 5:**
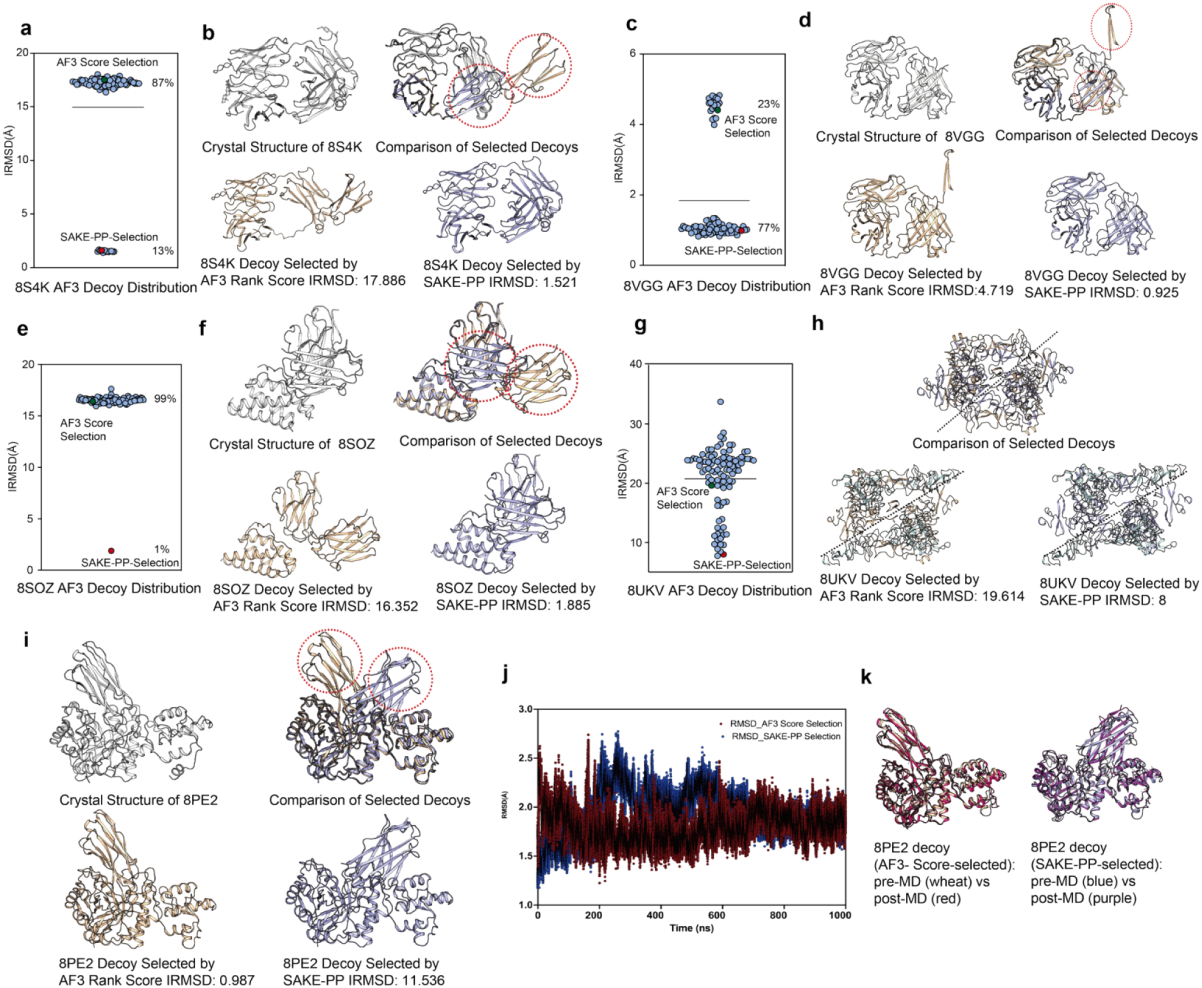
(a, c, e, g) Scatterplots of iRMSD for all 100 AF3-generated decoys of PDB IDs 8S4K, 8VGG, 8SOZ, and 8UKV. The symbol highlighted in red marks the decoy chosen by SAKE-PP; the blue symbol marks that chosen by the AF3 ranking score. Percentages indicate the fraction of decoys with equal or lower iRMSD than the AF3 ranking score-selected model. (b, d, f, h) Structural overlays. For each target, the crystal structure is shown in gray. Decoys selected by AF3 ranking score (tan) and SAKE-PP (slate-blue) are superposed; red dashed circles highlight regions where the two selections differ markedly. Corresponding iRMSD values are listed beneath each panel. (i) Same overlay for 8PE2, where SAKE-PP intentionally chose a more dissimilar starting pose (iRMSD = 11.536 Å) to test sampling robustness. (j) All-atom MD refinement of the AF3 ranking score-selected (wheat) and SAKE-PP-selected (blue) 8PE2 decoys: backbone RMSD vs simulation time over 1 μs. Post-MD conformations are colored red (AF3) and purple (SAKE-PP). (k) Final MD snapshots of the two 8PE2 decoys, showing that the SAKE-PP start converges toward the experimental structure while the AF3 ranking score starts to drift away.

Moreover, randomly sampling 10 decoys per group confirms a systematic preference: deviated decoys receive higher AF3 scores (mean 0.8572, s.d. 0.005) than reasonable decoys (mean 0.8399, s.d. 0.004). Occasional reasonable outliers (e.g., iRMSD 1.495 Å at AF3 score 0.8501) further indicate limited sensitivity to subtle binding-site deviations. By contrast, the top seven SAKE-PP-ranked conformations all fall within the reasonable range.

Similar bias is observed in 8VGG and 8SOZ. For 8VGG, although 77% of decoys are near-native (iRMSD <2 Å), AF3 can still rank an erroneous outlier (iRMSD ≥4 Å; 23%) highest, driven by a solvent-exposed extended loop/β-strand whose position is difficult to model. For 8SOZ, the near-native model (iRMSD 1.885 Å) represents only 1% of the ensemble and ranks seventh by AF3, while the remaining 99% cluster around 16.5 Å; neighboring AF3-ranked decoys around the best model also have iRMSD ≈ 16.5 Å, consistent with an anomalous domain-level deviation. SAKE-PP robustly recovers the near-native conformation (Figure 5e) and assigns a low predicted error (1.885 Å) to it, while scoring all other highly deviated decoys above 4 Å, demonstrating its value as a complementary scoring function when AF3 ranking fails under strong structural heterogeneity.

#### 3.2.3 Failure Cases

Although SAKE-PP generally performs well, we observed a subset of high-ranking failures. Closer inspection indicates that these errors primarily stem from pose selection rather than incorrect local folding.

In the 8PE2 evaluation, the SAKE-PP-selected decoy (iRMSD = 11.536 Å) is globally homologous to the AF3-selected structure (iRMSD = 0.987 Å).^33^ Despite a large Cα-RMSD, both models align closely in the central flexible loop and C-terminal β-sheet region. The key discrepancy is a mirror-like reorientation of the β-sheet domain (residues 444–561), with sheet-plane normals differing by ∼130° while preserving the fold, intrasheet hydrogen-bond network, and local backbone geometry, underscoring the intrinsic stability of this motif.

To probe physical stability, each decoy was solvated (TIP3P, 0.15 M NaCl) and simulated for 1 μs with Amber ff14SB at 300 K and 1 atm. The potential energy converged within 200 ns to approximately −8.0 × 10^5^ kJ mol^–1^ and fluctuated within ±5 × 10^3^ kJ mol^–1^. Across trajectories, secondary-structure content varied by ≤±2%, hydrogen-bond patterns remained stable, SASA changed by <8%, and B-factor profiles showed that the most mobile regions coincide between the SAKE-PP- and AF3-selected models, indicating similar intrinsic flexibility. Furthermore, we provide MD structural snapshots at 10%–90% of the trajectory for both systems (Figures S4–S23). These visual observations are further supported by quantitative Cα and all-atom RMSD analysis of Chain B (residues 443–563), the ligand chain positioned differently in the AF3- and SAKE-PP-selected models (Figures S24 and S25). The AF3 system fluctuates between 0.898 and 1.810 Å (mean 1.43 ± 0.28 Å), whereas the SAKE-PP system remains confined to 1.092–1.503 Å (mean 1.34 ± 0.15 Å) and converges toward ∼1.1 Å in the second half of the simulation. Additionally, we provide a more intuitive radar plot (Figure S26) to visualize the RMSD profiles of both systems across all time points. Both the AF3- and SAKE-PP-selected decoys remain structurally stable throughout the simulation. The SAKE-PP-selected decoys shows smaller conformational variance, suggesting closer proximity to a more favorable local minimum despite its larger initial iRMSD.

We next examined three representative failures (8XYZ, 8YF2, and 8K0E) where SAKE-PP deviates by >16 Å in iRMSD while AF3 remains within 2 Å (Figure S27). In all three, backbone folds are nearly identical, suggesting the dominant error is the relative placement of the partner chain in the binding pocket. For 8XYZ and 8YF2, SAKE-PP tends to drive the ligand into a hydrophobic pocket formed by a contracted loop, prioritizing tight packing and geometric complementarity, whereas AF3 favors poses closer to biologically plausible configurations in signaling proteins. For 8K0E, both methods insert the ligand into a deep pocket, but SAKE-PP selects a more compact pocket defined by closely packed β-strands, while AF3 selects a pocket supporting broader contacts.

Because purely geometric analyses may miss the energetic basis of binding, we further performed MD-based binding-energy calculations. Across all systems, SAKE-PP-selected conformations consistently showed more favorable binding energies than AF3 ranking score-selected ones (Table 2), including 8K0E (−75.77 vs −71.41 kcal/mol), 8YF2 (−50.99 vs −26.39 kcal/mol), and 8XYZ (−30.97 vs −29.39 kcal/mol). These results suggest that the advantage of SAKE-PP is not only geometric but also energetic.

**Table 2:** Comparison of Predicted Binding Free Energies Using AF3 Ranking Score and SAKE-PP Methods

PDB ID	Method	Binding Free Energy (kcal/mol) ↓
8K0E	AF3 ranking score	–71.41
8K0E	SAKE-PP	–75.77
8YF2	AF3 ranking score	–26.39
8YF2	SAKE-PP	–50.99
8XYZ	AF3 ranking score	–29.39
8XYZ	SAKE-PP	–30.97

To be more specific, residue-level analysis indicates that this improvement is driven by the quality, rather than simply the number, of interface contacts. In 8K0E, although AF3 identifies more interface residues within 10 Å (144 vs 116; 113 shared), its additional residues contribute only −1.78 kcal/mol in total and include several repulsive sites, such as 467GLU, 644ASP, 632ASP, and 636MET, each contributing more than +1 kcal/mol, as shown in Figure S30a. By contrast, SAKE-PP yields a net 4.36 kcal/mol stronger binding and strengthens the contributions of shared hotspot residues, including 92ARG, 236GLN, 61TRP, 55TRP, and 46ARG, each improved by >1 kcal/mol through π–π, π–cation, π–alkane, and hydrogen-bonding interactions (Table S3 and Figure S30b). Although both methods identify 35 hotspot residues, SAKE-PP more effectively reinforces their interfacial coupling.

A similar trend can be observed in 8YF2. SAKE-PP identifies more interface residues than AF3 (86 vs 59), with the additional 27 residues contributing −9.21 kcal/mol in total (Figure S29). Among them, 17TYR and 12ARG make particularly strong favorable contributions (4.44 and 3.38 kcal/mol, respectively). At the same time, SAKE-PP also amplifies the contributions of shared residues, for example improving 83LYS from −2.36 to −7.05 kcal/mol and 87TYR from 0.21 to −3.28 kcal/mol, consistent with enhanced electrostatic and π-system interactions (Table S4). Together, these results support that SAKE-PP preferentially selects conformations with more favorable energetic organization at the binding interface.

Together, these cases show that SAKE-PP failures are mainly pose-selection errors under strong structural heterogeneity, yet its scoring correlates well with binding energetics and preferentially selects conformations with more favorable affinities.

### 3.3 Antibody: SAKE-PP Benchmark beyond the Training Distribution

To probe how well SAKE-PP performs outside the distribution on which it was trained, we designed an antibody–antigen benchmark. This setting is deliberately challenging: the model was optimized only on a generic set of dimeric protein–protein interactions and has never seen multichain antibody–antigen complexes during training. Its performance here therefore provides a direct measure of its ability to generalize to entirely new protein classes.

For the benchmark, we began with the SAbDab data set curated by Fang et al. Redundancy was removed, chains shorter than 20 residues were discarded, and only entries containing complete antibody–antigen pairs were retained.^34,35^ After these filters, 139 unique antibody–antigen complexes remained and were used for evaluation. For each complex, AF3 first generated 200 decoys, and this decoy set was then reranked by SAKE-PP (Figure 6a). Globally, the Pearson correlation between score and iRMSD is −0.2886 for the AF3 ranking score, indicating a pronounced negative trend, whereas SAKE-PP improves the correlation to +0.1116, a gain of almost +0.40. On a per-complex basis, SAKE-PP outperforms the AF3 ranking score in 106 of 139 cases (76.3%), while the AF3 ranking score is superior in only 33 cases (23.7%). Strikingly, 70 complexes (>50% of the test set) flip from negative to positive correlation when rescored with SAKE-PP, underscoring the method’s stronger interface-aware ranking capability.

**Figure 6:**
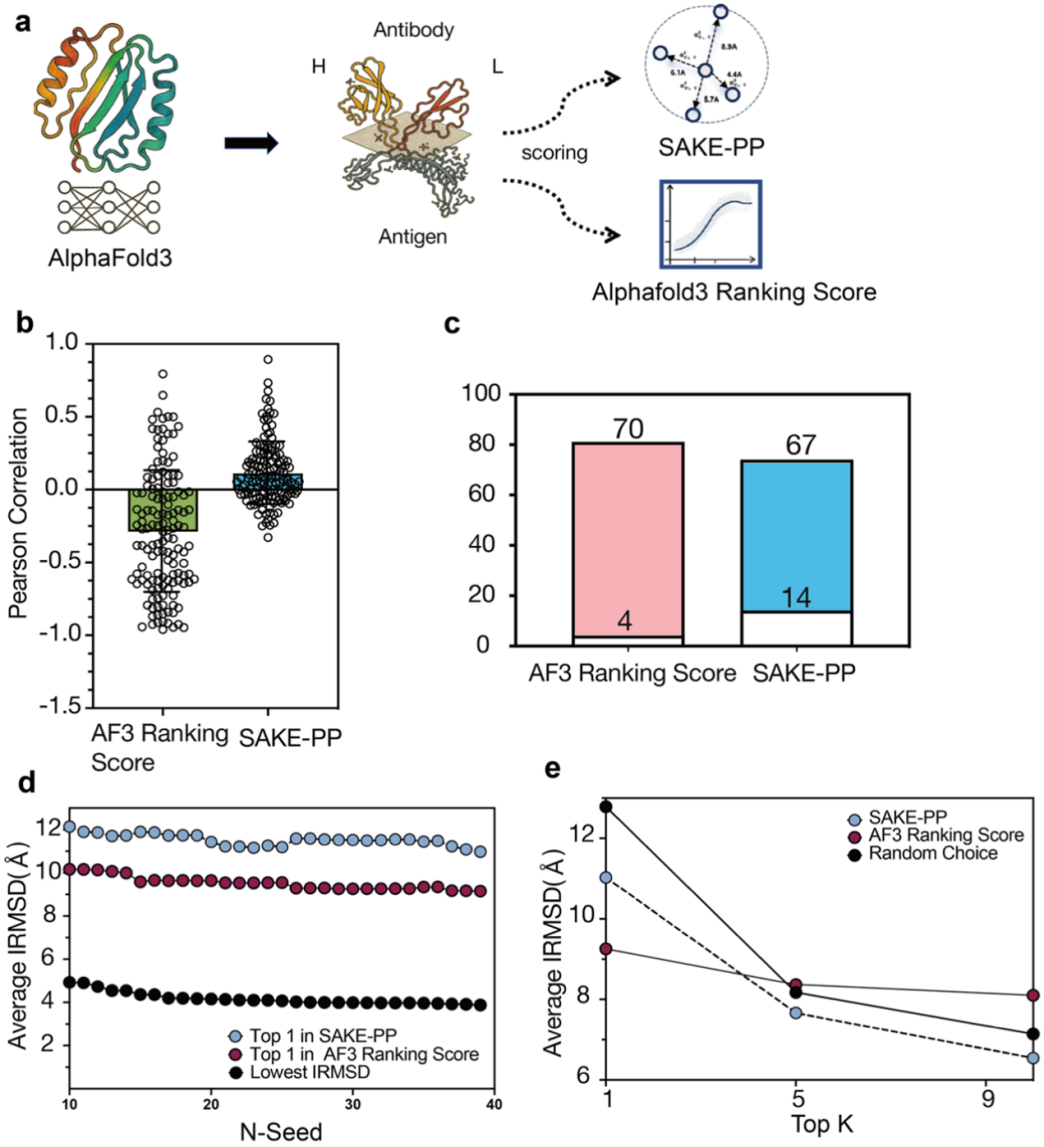
Antigen benchmark: SAKE-PP versus the AF3 ranking score. (a) Workflow. AF3 first generates 100 decoys for each heavy-light-chain antibody bound to its antigen; the decoys are then rescored by SAKE-PP (blue) or by the native AF3 ranking score (gray). (b) Pearson correlations (*r*) between predicted score and iRMSD for every complex (*n* = 74). Each point is a single complex; boxes indicate the interquartile range. AF3 correlations cluster around negative values (green), whereas SAKE-PP correlations are predominantly positive (blue). (c) Count of complexes with positive versus negative correlation. AF3 ranking score yields only 4 positive and 70 negative cases, while SAKE-PP reverses the trend with 67 positive and 14 negative cases. (d) Seed-selection experiment. As the number of seeds (*N*) increases, the average iRMSD of the single best seed chosen by SAKE-PP (blue) or by AF3 ranking score (purple) is compared with the theoretical optimum—the lowest-iRMSD decoy in the pool (black). (e) Average iRMSD achieved when retaining the top-*k* decoys (*k* = 1, 5, 9). SAKE-PP (blue) consistently outperforms AF3 ranking score (purple) and a random baseline (black dashed).

Although SAKE-PP trails the native AF3 ranker by a single complex in the “pick-one” headline metric (the AF3 ranking score selects the lower-iRMSD top-1 decoy in 70 cases, SAKE-PP in 69), a closer look at the difficult subset reveals a very different picture. We isolated the 18 antibody–antigen complexes for which the AF3 ranking score assigns a high confidence score (ranking score >0.75) yet the corresponding decoy is clearly wrong (iRMSD >4 Å). In other words, these are the instances where the AF3 score is most misleading. Within this challenging cohort, SAKE-PP outperforms the AF3 ranking score in 14 out of 18 cases, correctly demoting the erroneous high-score decoys and surfacing nearer-native alternatives.

Abramson et al. (2024) have argued that attaining peak accuracy with AF3 in antibody–antigen systems require generating and reranking a very large number of seed decoys, which incurs substantial computational overhead.^15^ To test this claim, we expanded each antibody–antigen decoy pool from 50 to 200 structures and retained only the top-ranked model according to either SAKE-PP or the native AF3 ranking score, while also recording the theoretical optimum (the lowest iRMSD in the pool). As shown in Figure 6d, the mean iRMSD of all three traces decreases as the number of seeds doubles, confirming that deeper sampling indeed increases the chance of encountering near-native poses. The AF3 ranking score average remains slightly better than that of SAKE-PP, a difference driven mainly by a few outlier complexes (e.g., 7XJ6, 7XJ8) where SAKE-PP makes large orientation errors, highlighting that the method can still be misled by extreme local heterogeneity.

From a downstream perspective, however, what matters most is the quality of the top-*k* candidates that will proceed to molecular-dynamics refinement or affinity maturation. We therefore compared the best iRMSD found within the top 1, top 5, and top 10 decoys out of 200 (Figure 6e) and benchmarked them against randomly chosen sets of the same size. Across all thresholds, SAKE-PP consistently outperforms the random baseline and converges to an average iRMSD of 6 Å for the top 10, whereas the AF3 ranking score lags behind random choice in the top-5 and top-10 scenarios, indicating that its native score struggles to enrich high-quality structures in large decoy pools. Taken together, while more extensive sampling benefits both methods in principle, SAKE-PP is markedly better at concentrating near-native decoys toward the very top of the ranking, delivering a smaller yet higher-quality candidate set that keeps subsequent MD simulations both reliable and computationally manageable.

It is important to emphasize that SAKE-PP was never trained on antibody–antigen complexes; it was optimized solely on a general protein–protein data set. The strong performance observed here, therefore underscores the model’s ability to generalize beyond its training distribution.

## 4 Conclusions and Outlook

We present SAKE-PP, a graph neural network for protein–protein interface evaluation that consistently outperforms AF3′s native ranking score across multiple benchmarks. While AF3 generates high-quality and diverse decoy ensembles, its confidence scores often fail to select the most biologically relevant, low-energy conformations. SAKE-PP improves iRMSD-based conformation selection by 13.75% relative to AF3, addressing this critical ranking bottleneck. This improvement arises from SAKE-PP’s physics-inspired design, which integrates spatial attention, equivariant kinetic updates, and Laplacian eigenvector–based directional encoding to more faithfully represent interfacial geometry. As a result, SAKE-PP better discriminates near-native from deviated conformations, even when both are present in the candidate set. SAKE-PP also exhibits strong generalization. In cross-data set evaluation and zero-shot antibody–antigen complexes, it exceeds AF3 ranking scores by up to *r* = 0.4, despite AF3 having been trained on substantial antibody–antigen data. This suggests that SAKE-PP captures transferable geometric principles of protein interfaces beyond data set-specific biases.

Finally, molecular dynamics and binding-energy analysis on 8PE2 indicate that SAKE-PP-selected conformations are thermodynamically favorable, even when less similar to the crystal pose, supporting its utility as a starting point for downstream tasks such as free-energy refinement and ligand design. These results motivate ensemble-based workflows and suggest extensions of SAKE-PP to larger assemblies and integration with adaptive sampling and free-energy methods for improved structure prediction and drug discovery.

Nevertheless, SAKE-PP can exhibit larger errors for inputs that lie far outside its training distribution, and purely geometry-driven scoring may sometimes deviate from biologically preferred poses in context-dependent crystal environments. In such cases, SAKE-PP may favor an interface that is more tightly packed and seemingly more energetically favorable, whereas AF3 may select a pose that is closer to the experimental structure or more compatible with functional constraints. These observations motivate future extensions that incorporate contextual priors (e.g., experimentally informed restraints, partner-specific interface preferences, or energetic/solvation terms) to better balance energetic plausibility, geometric complementarity, and biological relevance.

## Supplementary Material



## Data Availability

All relevant data are provided in the Supporting Information. The code could be found in the following: https://github.com/yxnyu/SakePP/.

## Notes

Complete PDB ID lists for the 176-complex 2024PDB test set and the 139-complex antibody–antigen benchmark; a detailed methodological description of SAKE-PP covering protein graph construction (Figure S1); cross-validation training dynamics and model generalization under the none-sampling strategy (Figure S2) and sampling strategy (Figure S3); molecular dynamics simulation protocols for 8PE2, including AF3-selected snapshots (Figures S4–S13), SAKE-PP–selected snapshots (Figures S14–S23), Cα RMSD analysis (Figure S24), multimetric RMSD comparison (Figure S25), and a radar plot of RMSD profiles (Figure S26); and binding free energy analysis for six heterogeneous case studies (8K0E, 8YF2, 8S4K, 8SOZ, 8VGG, 8XYZ), with iRMSD scatterplots and structural overlays (Figure S27), predicted binding free energies (Table S2), interface hotspot comparisons (Figures S28–S31), and per-residue ΔΔ *G* decomposition tables (Tables S2–S6) (PDF)
