# Supplementary material for: SAKE-PP: A Spatial-Attention Equivariant Network for Accurate Ranking of Protein–Protein Interaction Models
Source: JACS Au. 2026 Apr 16;6(5):2846–56. doi: 10.1021/jacsau.6c00166 (PMC13213501; doi:10.1021/jacsau.6c00166)
Supplement: Supplementary file 1 [file au6c00166_si_001.pdf]

Supporting Information

SAKE-PP: A Spatial-Attention Equivariant Network for Accurate Ranking of Protein–Protein Interaction Models

Yuzhi Xu,[1,2,†] Wei Xia,[1,2,†] Chao Zhang,[3] Xinxin Liu,[4] Cheng-Wei Ju,[5] Xuhang Dai,[2] Pujun Xie,[6] Yuanqing Wang,[2,*] Guangyong Chen,[4,*] and John Z.H. Zhang[1,2,3,7,*]

[1]NYU-ECNU Center for Computational Chemistry, NYU Shanghai, Shanghai 200126, China

[2]Department of Chemistry, New York University, New York, New York 10003, United States

[3]Faculty of Synthetic Biology, Shenzhen University of Advanced Technology, Shenzhen 518107, China

[4]Hangzhou Institute of Medicine, Chinese Academy of Sciences, 150 Dongfang Street, Xiasha, Qiantang District, Hangzhou, Zhejiang 310018, China

[5]Pritzker School of Molecular Engineering, The University of Chicago, Chicago, Illinois 60615, United States

[6]Department of Biochemistry and Molecular Pharmacology, New York University Grossman School of Medicine, New York, NY 10016, USA

[7]Collaborative Innovation Center of Extreme Optics, Shanxi University, Taiyuan 030006, China

[†]These authors contributed equally and should be regarded as co-first authors.

[*]Correspondences: john.zhang@nyu.edu, gychen@link.cuhk.edu.hk, wangyq@umich.edu

1 2024PDB Test-Set PDB IDs

8PE1, 8SW5, 8S61, 8XYZ, 8K0E, 9G5K, 9AZ4, 8YF2, 8YFT, 8RT9, 8W6B, 8PE2, 8S5W, 8RQC, 8QU7, 8K3A, 9FZD, 8EPH, 8SVE, 8S5U, 8RY2, 9B75, 8K4Q, 8IDA, 8R38, 8UW8, 8VEG, 8VTP, 9BHW, 8S1X, 8S4U, 9AXN, 8S4H, 8SJB, 8VGE, 8SFX, 8RTX, 8YFE, 8G4K, 8PMW, 8QVX, 8BOP, 8X7N, 9AXQ, 8WT4, 8RAJ, 9FZC, 8PUT, 8WZO, 8W6A, 8VVB, 8P6H, 8RPP, 8XF2, 8VK8, 8YSH, 8JZT, 8UMC, 8K9Z, 8FJ4, 8WBZ, 8XUP, 9GRQ, 9F98, 8X3G, 8VLT, 8QC1, 8QZ4, 9FDJ, 8GJR, 9G2A, 8SUY, 9FCJ, 9B76, 9FZK, 8Z5D, 8FRI, 8WEO, 8RO6, 9IY0, 8VK9, 9GNY, 9AXO, 8V00, 8Z8V, 8F2Z, 8RO9, 9D65, 9D6J, 8RO8, 8VGF, 9IUZ, 8EPC, 9D6K, 9D6M, 8Z5F, 9ATO, 9FW2, 8TYX, 8QXC, 9FZ4, 8YJ5, 8POX, 8RO7, 9BD3, 9BIY, 8F6S, 8QZ3, 8IK6, 8UG2, 8Q6Q, 9ASS, 8S0N, 8WIX, 8VJM, 8P2T, 8WPL, 9AVT, 8WK1, 8SOW, 9FEF, 8GDT, 8YOD, 8J09, 8RD3, 8VU4, 8VU1, 8VUC, 8WHZ, 8SFV, 8WBY, 8W70, 8KB8, 9GP2, 8ROH, 8WZN, 8SFS, 9B44, 9DP6, 8X77, 8PDC, 8WKS, 9B74, 8OWS, 8PEQ, 8WFH, 9F18, 8VTR, 8SLC, 8U70, 8TE7, 8IGD, 8ZJF, 8SG3, 8Z8M, 9ATP, 8PMZ, 8UX9, 8IKV, 8QJ0, 8PU3, 8T19, 8WFM, 8RZ0, 9IU1, 8VGG, 8W90, 8K33, 8JI9, 8XN2, 8XN5, 8Y0Y, 8UKV, 8SOZ, 9FO7, 8S4K

Most of these proteins were released after June 2024, ensuring they fall strictly outside the AlphaFold3 training window. Although we did not perform explicit homology filtering, this omission can only confer a limited advantage to AF3 ranking score, because the SAKE-PP training data are already subsumed within AF3 ranking score.

2 Antibody–Antigen Dataset PDB IDs

7QUH, 7ST5, 7TRH, 7U9E, 7UA2, 7UJA, 7UMN, 7UOW, 7UXL, 7WN2, 7WNB, 7XCZ, 7XDA, 7XDK, 7XDL, 7XEG, 7XEI, 7XIK, 7XIL, 7XJ6, 7XJ8, 7XJ9, 7XJF, 7XRZ, 7XS8, 7XSA, 7XSC, 7Y8J, 7YD1, 7YDS, 7YK4, 7YRU, 7YUE, 7YV1, 7ZOZ, 7ZQT, 8A44, 8A96, 8A99, 8AHN, 8AV9, 8BCZ, 8BSE, 8BSF, 8BYU, 8C3V, 8CIM, 8CT6, 8CWI, 8CWJ, 8CWK, 8CZ8, 8CZZ, 8D7E, 8D9Y, 8D9Z, 8DA0, 8DA1, 8DB4, 8DE4, 8DN6, 8DN7, 8DNN, 8DTO, 8DUZ, 8DWW, 8DWY, 8DY1, 8DY5, 8DYX, 8DZ3, 8DZV, 8E1G, 8E1M, 8E6J, 8E6K, 8EE0, 8EE1, 8EK1, 8EKA, 8EL2, 8ELO, 8ELP, 8ELQ, 8EOO, 8EPA, 8F0H, 8F60, 8F6L, 8F6O, 8FAX, 8FG0, 8G30, 8G3M, 8G3N, 8G3O, 8G3P, 8G3Q, 8G3R, 8G3V, 8G3Z, 8GB6, 8GB7, 8GB8, 8GNK, 8GS9, 8GTP, 8GTQ, 8H07, 8HC4, 8HC5, 8HEB, 8HEC, 8HED, 8HHX, 8HHY, 8HN6, 8HN7, 8I5H, 8I5I, 8IB1, 8IUK, 8IUM, 8IW9, 8OL9, 8SAQ, 8SAR, 8SAS, 8SAV, 8SAW, 8SAX, 8SAY, 8SB0, 8SB1, 8SB2, 8SB3, 8SB5, 8SCX, 8SMT

3 Methods Details in SAKE-PP

Protein Structure Representation

We represent a protein structure as a graph $\mathcal{G} = (\mathcal{V}, \mathcal{E})$, where each node $v \in \mathcal{V}$ corresponds to one residue in the protein backbone, and edges $\mathcal{E}$ capture their spatial proximity and biochemical relationships. Let $|\mathcal{V}| = N$ denote the number of residues (nodes), and let $\mathcal{E} \subseteq \{(u, v) \mid u, v \in \mathcal{V}\}$ be the edge set.

Node Representation

For each residue $v \in \mathcal{V}$, we collect both *spatial* and *chemical* features:

Spatial Features.

- **Cα coordinates**: We treat the entire protein–ligand complex (or multi-chain complex) as a single structure and extract all Cα atoms from the backbone. Each Cα atom is indexed by a node v, whose 3D position is given by $\mathbf{x}_v \in \mathbb{R}^3$ (i.e., the (x, y, z) coordinates). These coordinates allow the model to capture the overall geometry of the protein complex.

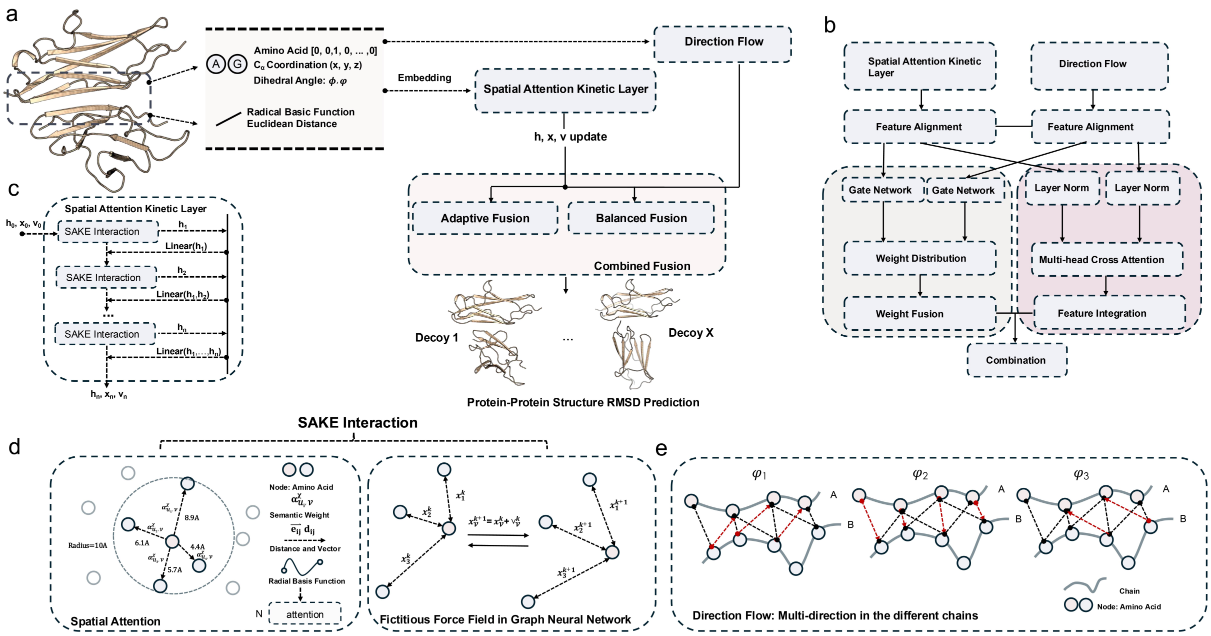

Figure S1: Workflow and architecture of SAKE-PP for protein–protein structure iRMSD prediction. (a) Workflow of SAKE-PP: Each protein complex is encoded using amino acid type, dihedral angles (ϕ, ψ), Cα coordinates (x, y, z), and Euclidean distance–based features within a 10 Å interface region. The representations are passed to both the Direction Flow and the Spatial Attention Kinetic Layer. Their outputs are integrated in the Combined Fusion module to predict a scalar iRMSD value. (b) Feature fusion mechanism: The two representation streams undergo feature alignment and are processed by gate networks, weight fusion, and multi-head cross attention to yield a unified latent representation. (c) Spatial Attention Kinetic Layer: Composed of stacked SAKE Interactions, each connected via residual links. Each SAKE layer updates node features $\mathbf{h}$, positions $\mathbf{x}$, and velocities $\mathbf{v}$ in an $E(n)$-equivariant manner. (d) SAKE Interaction Module: Integrates spatial attention and a fictitious force field for joint node and coordinate updates. The spatial attention mechanism combines radial basis encoding of pairwise distances with edge-based semantic attention, capturing local geometric dependencies. A mixed attention score $\alpha_{uv}^{X\times H}$ modulates neighborhood aggregation. Position updates are coupled with learned velocity vectors that evolve under a physics-inspired force field: $\mathbf{x}_v^{(k+1)} = \mathbf{x}_v^{(k)} + \mathbf{v}_v^{(k)}$, $\mathbf{v}_v^{(k+1)} = \phi^{v\to V}(\mathbf{h}_v^{(k)})\mathbf{v}_v^{(k)} + \sum_{u\in\mathcal{N}(v)} \lambda_i(\mathbf{h}_{e_{uv}}^{(k)})\, f(\vec{\mathbf{e}}_{uv}^{\,k})$. (e) Direction Flow: Captures multi-directional spatial interactions across protein chains using chain-aware angular relationships (φ_1, φ_2, φ_3) to guide geometric feature propagation.

- **Backbone dihedral angles**: For each residue v, we calculate the main-chain dihedral angles (ϕ_v, ψ_v) following the standard protein geometry definition. In rare cases where numerical issues arise (e.g., a tiny subset of angles failing to compute properly), we assign a small default value (e.g., 10^{-5}) to avoid undefined behaviors in subsequent computations.

- **Angle-derived embedding**: We adopt the angle-derived embedding $\mathbf{a}_v \in \mathbb{R}^3$ to convert dihedral angles into a 3D direction vector. Formally,

$$\mathbf{a}_v \;=\; \text{angle2vec}(\phi_v, \psi_v) \;=\; \big[\sin(\phi_v)\cos(\psi_v),\ \sin(\phi_v)\sin(\psi_v),\ \cos(\phi_v)\big]. \tag{1}$$

Chemical Features.

- **Residue type encoding**: We represent each canonical amino acid with a one-hot vector or learned embedding, $\mathbf{r}_v \in \mathbb{R}^{20}$.
- **Chain identifier**: Each residue v is associated with a chain identifier $c_v \in \mathbb{N}$, which indicates which protein chain the residue belongs to (e.g., chain A, chain B, etc.).
- **Cα flag**: We include a binary flag $f_v \in \{0, 1\}$ to specify whether the entry corresponds to a Cα atom (main-chain α carbon).

By concatenating the chemical features above with the spatial features described previously, we arrive at the *complete node feature* vector. Specifically, we form

$$\mathbf{h}_v = \left[\mathbf{r}_v \ \oplus \ \mathbf{a}_v \ \oplus \ \mathbf{x}_v\right] \ \in \ \mathbb{R}^{26}, \tag{2}$$

where $\oplus$ denotes vector concatenation. In this construction,

- $\mathbf{r}_v \in \mathbb{R}^{20}$ encodes the residue identity,
- $\mathbf{a}_v \in \mathbb{R}^3$ (angle-derived embedding) represents dihedral angles,
- $\mathbf{x}_v \in \mathbb{R}^3$ stores the Cα coordinates.

Hence, each residue v is ultimately represented by a 26-dimensional feature vector that integrates both chemical and spatial information. This design aligns with our code's practice of combining residue type, angle-based features, and 3D coordinates into a single node embedding.

Edge Representation

To fully capture inter-residue interactions, we define both the *edge set* $\mathcal{E}$ and the *edge features* $\mathbf{f}_{e_{uv}}$ in one integrated procedure. Specifically, we first construct edges based on complementary criteria, then compute a rich set of geometric and biochemical descriptors for each edge.

Edge Construction. We adopt two strategies to determine whether an edge should exist between residues u and v:

1. **Contact-based rule**:

$$\mathcal{E}_d \ = \ \left\{ (u, v) \ \mid \ \|\mathbf{x}_u - \mathbf{x}_v\| < d_0, \ u \neq v \right\}, \tag{3}$$

where $d_0 = 10\,\text{Å}$ is a typical cutoff for identifying residue-residue contacts.

2. **k-Nearest Neighbors (kNN)**: For each residue v, we pick $k = 30$ closest neighbors by Euclidean distance on Cα coordinates:

$$\mathcal{N}_k(v) \;=\; \mathrm{argsort}_u\big(\|\mathbf{x}_u - \mathbf{x}_v\|\big)\_1 : k, \quad \mathcal{E}_k \;=\; \bigcup_{v \in \mathcal{V}} \big\{(v,u) \;\mid\; u \in \mathcal{N}_k(v)\big\}. \tag{4}$$

We then merge these sets to form the final edge set:

$$\mathcal{E} \;=\; \mathcal{E}_d \,\cup\, \mathcal{E}_k, \tag{5}$$

ensuring that both long-range contacts (within d_0) and local neighbors ($k = 30$) are included.

Edge Features. Once the edge set $\mathcal{E}$ is established, we compute an *edge feature vector* $\mathbf{f}_{e_{uv}}$ for each $(u, v) \in \mathcal{E}$. This vector encodes geometric and biochemical information essential for downstream message passing. Concretely, we combine:

- **Displacement vector and Distance**:

$$\vec{\mathbf{e}}_{uv} \;=\; \mathbf{x}_v - \mathbf{x}_u, \qquad l_{uv} \;=\; \big\|\vec{\mathbf{e}}_{uv}\big\|. \tag{6}$$

- **Direction**:

$$\hat{\mathbf{e}}_{uv} \;=\; \frac{\vec{\mathbf{e}}_{uv}}{l_{uv} + \varepsilon}, \tag{7}$$

  where ε is a small positive constant (e.g., 10^{-6}) to avoid division by zero.

- **Radial Basis Expansion**: *I*n our implementation, we expand the raw distance l_{uv} via a set of radial basis functions:

$$\mathrm{RBF}(l_{uv}) \;=\; \Big[\exp\!\Big(-\frac{\big(l_{uv} - \mu_i\big)^2}{2\sigma^2}\Big)\Big]_{i=1}^{N_{\mathrm{rbf}}}, \tag{8}$$

  where $\{\mu_i\}_{i=1}^{N_{\mathrm{rbf}}}$ are centers evenly spaced in $[0, d_0]$, σ is a bandwidth, and we fix $N_{\mathrm{rbf}} = 16$. This 16-channel representation provides a smooth and more expressive encoding of inter-residue distances.

- **Source and Target Residue Types**: For each edge (u, v), we can include the amino acid encodings $\mathbf{r}_u, \mathbf{r}_v \in \mathbb{R}^{20}$, thereby allowing the model to learn pairwise interaction patterns (e.g., hydrophobic vs. hydrophobic).

- **Chain Interaction Flag**:

$$\delta_{uv} \;=\; \mathbb{I}\big[c_u = c_v\big], \tag{9}$$

which indicates whether residues u and v belong to the same chain. In interface-focused tasks, one might specifically highlight the inter-chain edges (i.e., $c_u \neq c_v$).

Hence, the final edge feature vector (concatenating all the above) is:

$$\mathbf{f}_{e_{uv}} \;=\; \big[l_{uv},\; \hat{\mathbf{e}}_{uv},\; \mathrm{RBF}(l_{uv}),\; \mathbf{r}_u,\; \mathbf{r}_v,\; \delta_{uv},\; \ldots\big]. \tag{10}$$

SAKE Interaction Layer: Spatial Attention and Kinetic Updates

In the previous work, we present a noval neural network named **SAKE** (*spatial attention kinetic network with E(n)- Equivariance*) layer aims to simultaneously update the features of *(i)* nodes (residue embeddings) and *(ii)* their 3D positions with E (n) -equivariance.[?] In the following, we outline the essential components of SAKE and explain how they integrate into a single framework.

Spatial Attention Definition. Given a node v with embedding $\mathbf{h}_v \in \mathbb{R}^C$ and position $\mathbf{x}_v \in \mathbb{R}^n$ in graph $\mathcal{G}$, the *spatial attention* ϕ^{SA} is designed to capture geometric relationships among neighbors $u \in \mathcal{N}(v)$:

$$\phi^{\mathrm{SA}}(v) \;=\; \mu\Big(\bigoplus_{i=1}^{N_\lambda}\Big\| \sum_{u\in\mathcal{N}(v)} \lambda_i(\mathbf{h}_{e_{uv}})\, f(\vec{\mathbf{e}}_{uv})\Big\|\Big), \tag{11}$$

where $\mathbf{h}_{e_{uv}}$ is the edge embedding, λ_i are attention weight functions (*i.e.*, small MLPs) mapping edge embeddings to scalars, and f is an E(n)-equivariant transformation on the displacement vector $\vec{\mathbf{e}}_{uv} = \mathbf{x}_v - \mathbf{x}_u$.

Edge Embedding Update. Prior to applying attention weights, each edge (u, v) is assigned an embedding $\mathbf{h}_{e_{uv}}$ that combines both chemical and geometric features:

$$\mathbf{h}_{e_{uv}}^{(k)} = \phi^e\Big(\mathbf{h}_u^{(k)} \oplus \mathbf{h}_v^{(k)} \oplus \|\vec{\mathbf{e}}_{uv}\| \oplus \mathrm{RBF}\big(\|\vec{\mathbf{e}}_{uv}\|\big) \odot f_r(\mathbf{h}_u^{(k)} \oplus \mathbf{h}_v^{(k)})\Big), \tag{12}$$

where RBF encodes distance into radial basis channels, and f_r is a small network that modulates the combined embeddings.

Mixed Attention. SAKE uses a combination of:

- A distance-based cutoff α_{uv}^X to smoothly suppress edges beyond a certain threshold d_0
- A semantic attention α_{uv}^H akin to GAT-style softmax weights that highlight edges with higher feature similarity

These are combined as:

$$\alpha_{uv}^{X\times H} = \frac{\alpha_{uv}^{X}\,\alpha_{uv}^{H}}{\sum_{w\in\mathcal{N}(v)}\alpha_{wv}^{X}\,\alpha_{wv}^{H}}. \tag{13}$$

Velocity and Position Updates. The position updates are coupled with velocity vectors:

$$\mathbf{v}_v^{(k+1)} = \phi^{v\to V}(\mathbf{h}_v^{(k)})\,\mathbf{v}_v^{(k)} \;+\; \mathbf{W}_v \sum_i \sum_{u\in\mathcal{N}(v)} \lambda_i(\mathbf{h}_{e_{uv}}^{(k)})\,f(\vec{\mathbf{e}}_{uv}^{\,k}), \quad \mathbf{x}_v^{(k+1)} = \mathbf{x}_v^{(k)} + \mathbf{v}_v^{(k)}. \tag{14}$$

Laplacian Eigenvector Directional Graph Neural Network

Laplacian Matrix and Eigenvectors. For an undirected graph $\mathcal{G}=(V,E)$, we define the graph Laplacian matrix $\mathbf{L}\in\mathbb{R}^{N\times N}$ either in the combinatorial form $\mathbf{L}=\mathbf{D}-\mathbf{A}$ or in the normalized form $\tilde{\mathbf{L}}=\mathbf{I}-\mathbf{D}^{-1/2}\mathbf{A}\,\mathbf{D}^{-1/2}$, where $\mathbf{A}$ is the adjacency matrix (with $A_{ij}=1$ if $(i,j)\in E$ and 0 otherwise) and $\mathbf{D}$ is the degree matrix ($D_{ii}=\sum_j A_{ij}$). The Laplacian is symmetric positive semidefinite, so it admits an eigendecomposition

$$\mathbf{L}\,\mathbf{U}=\mathbf{U}\,\mathbf{\Lambda} \quad \text{or} \quad \tilde{\mathbf{L}}\,\mathbf{U}=\mathbf{U}\,\mathbf{\Lambda},$$

where $\mathbf{U}=[\phi_0\,\phi_1\,\ldots\,\phi_{N-1}]$ is an orthonormal basis of eigenvectors, and $\mathbf{\Lambda}=\mathrm{diag}(\lambda_0,\lambda_1,\ldots,\lambda_{N-1})$ are the corresponding eigenvalues (sorted as $0\le\lambda_0\le\lambda_1\le\ldots$). The first eigenvector ϕ_0 associated with $\lambda_0=0$ is the constant vector; The trivial eigenvector ϕ_0 associated with the constant vector is discarded, and the subsequent k *nontrivial* eigenvectors $\{\phi_1,\ldots,\phi_k\}$ are retained to capture more meaningful structural properties of G.

Laplacian Eigenvector and directional aggregators in Laplacian Eigenvector Directional Graph Neural Network Let $\mathcal{G}=(V,E)$ be the input graph with $|V|=N$ nodes and $|E|=M$ edges. Denote the node features by $\mathbf{X}\in\mathbb{R}^{N\times d_{\text{in}}}$ and the edge features by $\mathbf{E}\in\mathbb{R}^{M\times d_e}$. We first map $\mathbf{X}$ to the hidden dimension d_{hid} via

$$\mathbf{H}^{(0)} = \mathrm{Dropout}\Big(\sigma\big(\mathbf{X}\,\mathbf{W}_{\text{in}}\big)\Big),$$

where $\mathbf{W}_{\text{in}}\in\mathbb{R}^{d_{\text{in}}\times d_{\text{hid}}}$ is a learnable matrix, and σ is the LeakyReLU activation function.

We compute the first $k=3$ Laplacian eigenvectors $\mathbf{\Phi}\in\mathbb{R}^{N\times 3}$, serving as position embeddings (PE). That is, each row $\mathbf{\Phi}_{i,:}$ stores the eigenvector values $(\phi_1(i),\phi_2(i),\phi_3(i))$ for node i.

Specifically, for an edge (i,j), let ϕ_k denote the kth Laplacian eigenvector. The directional difference $F_{i,j}=\phi_k(j)-\phi_k(i)$ encodes how node j differs from node i in the kth eigenvector dimension. Two specialized aggregators, *directional average* and *directional derivative*, utilize $|F_{i,j}|$ or $F_{i,j}$ respectively to weigh neighbor features $\mathbf{h}_j$. In our work, these directional aggregators are combined with standard ones such as `mean`, `sum`,

and `max`, along with degree-based *scalers* (`identity`, `amplification`, `attenuation`), to capture both global and local structures. Hence, DGNConv offers richer geometric expressiveness than purely isotropic graph convolutions.

Laplacian Eigenvector Directional Graph Neural Network Layers. For each layer $\ell = 1, \ldots, L$, we apply a Laplacian Eigenvector Directional Graph Neural Network module, denoted by $\mathcal{F}^{(\ell)}$, which takes the graph $(\mathcal{G}, \mathbf{H}^{(\ell-1)}, \mathbf{E}, \mathbf{\Phi})$ as input. Inside $\mathcal{F}^{(\ell)}$, we use five aggregators: `mean`, `max`, `sum`, `dir1-av`, `dir1-dx`, and three scalers: `identity`, `amplification`, `attenuation`. Combining each aggregator with each scaler yields multiple feature transforms that are concatenated and projected back to d_{hid}. We then apply LeakyReLU and a dropout with rate p (e.g., $p = 0.15$). The output of layer ℓ is

$$\mathbf{H}^{(\ell)} = \text{Dropout}\Big(\sigma\big(\mathcal{F}^{(\ell)}(\mathcal{G},\, \mathbf{H}^{(\ell-1)},\, \mathbf{E},\, \mathbf{\Phi})\big)\Big).$$

After stacking L layers of DGNConv, we obtain

$$\mathbf{H}^{(L)} \in \mathbb{R}^{N \times d_{\text{hid}}},$$

which we map to the output dimension d_{out} via a linear projection:

$$\mathbf{Y} = \mathbf{H}^{(L)}\, \mathbf{W}_{\text{out}} \;\in\; \mathbb{R}^{N \times d_{\text{out}}}.$$

Here, $\mathbf{W}_{\text{out}} \in \mathbb{R}^{d_{\text{hid}} \times d_{\text{out}}}$ is a learnable parameter matrix. The returned node-level output $\mathbf{Y}$ thus has one row per node, each row being a d_{out}-dimensional feature.

SAKE-PP: Integrating SAKE and Laplacian Eigenvector Directional GNN

SAKE layers update both the node features and their 3D coordinates/velocities. We initialize the node features via a learnable linear map and optional activation/dropout,

$$\mathbf{h}^{(0)}_{\text{SAKE}} = \text{Dropout}\Big(\sigma\big(\mathbf{h}\, \mathbf{W}_{\text{init}}\big)\Big),$$

where $\mathbf{W}_{\text{init}} \in \mathbb{R}^{d_h \times d_{\text{SAKE}}}$. Then each SAKE Interaction Layer applies the spatial attention and kinetic updates (11–14), producing

$$(\mathbf{h}^{(\ell)}_{\text{res}},\, \mathbf{x}^{(\ell)},\, \mathbf{v}^{(\ell)}) = \text{SAKE\_Layer}\big(\mathbf{h}^{(\ell-1)}_{\text{SAKE}},\, \mathbf{x}^{(\ell-1)},\, \mathbf{v}^{(\ell-1)},\, \texttt{pairlist}\big).$$

Each residual node embedding $\mathbf{h}_{\text{res}}^{(\ell)}$ is then projected via an additional linear layer and concatenated with previous features, *e.g.*,

$$\widetilde{\mathbf{h}}_{\text{res}}^{(\ell)} = \mathbf{h}_{\text{res}}^{(\ell)} \mathbf{W}_{\text{add}}^{(\ell)}, \quad \mathbf{h}_{\text{SAKE}}^{(\ell)} = \text{ConcatMLP}\Big(\big[\mathbf{h}_{\text{SAKE}}^{(0)}, \ldots, \widetilde{\mathbf{h}}_{\text{res}}^{(\ell)}\big]\Big),$$

where ConcatMLP denotes a learned concatenation transform followed by batch normalization, activation, and dropout. After the final SAKE Interaction Layer (say $\ell = L_{\text{SAKE}}$), we obtain updated node embeddings $\mathbf{h}_{\text{SAKE}} \in \mathbb{R}^{N \times d_{\text{SAKE}}}$, as well as new positions/velocities $(\mathbf{x}, \mathbf{v})$.

In parallel, original node features $\mathbf{h}$ and the edge features $\mathbf{E}$ (together with the graph structure $\mathcal{G}$) are feeded into the Laplacian Eigenvector Directional GNN (Sec. 3). This module, denoted as `dgn`, outputs another node-level representation:

$$\mathbf{h}_{\text{DGN}} = \texttt{dgn}(\mathcal{G}, \mathbf{h}, \mathbf{E}).$$

Internally, `dgn` uses multiple DGNConv layers, each exploiting both standard and directional aggregators (e.g. `dir1-av`, `dir1-dx`) with degree-based scalers, ultimately producing $\mathbf{h}_{\text{DGN}} \in \mathbb{R}^{N \times d_{\text{DGN}}}$.

We then concatenate the SAKE output $\mathbf{h}_{\text{SAKE}}$ and the DGN output $\mathbf{h}_{\text{DGN}}$ along the feature dimension:

$$\mathbf{h}_{\text{combined}} = \big[\mathbf{h}_{\text{SAKE}}, \mathbf{h}_{\text{DGN}}\big] \in \mathbb{R}^{N \times (d_{\text{SAKE}} + d_{\text{DGN}})}.$$

An additional learnable transformation `combined_processing` is applied, which may be a linear layer or a small MLP with activation:

$$\mathbf{h}_{\text{combined}} = \texttt{combined\_processing}\big(\mathbf{h}_{\text{combined}}\big).$$

To obtain a graph-level representation, we perform global pooling over the node dimension:

$$\mathbf{u}_{\text{mean}} = \text{mean}\big(\mathbf{h}_{\text{combined}}\big), \quad \mathbf{u}_{\text{max}} = \max\big(\mathbf{h}_{\text{combined}}\big), \quad \mathbf{u}_{\text{sum}} = \text{sum}\big(\mathbf{h}_{\text{combined}}\big).$$

Here, mean, max, sum are taken node-wise (and separately within each subgraph if using a batched graph). We concatenate the three pooled vectors,

$$\mathbf{u}_{\text{graph}} = \big[\mathbf{u}_{\text{mean}}, \mathbf{u}_{\text{max}}, \mathbf{u}_{\text{sum}}\big],$$

and finally project to the desired scalar output (or any target dimension) via

$$\mathbf{y}_{\text{out}} = \mathbf{u}_{\text{graph}}\,\mathbf{W}_{\text{out}} \;\in\; \mathbb{R}^1 \quad \text{(or the appropriate output size).}$$

Thus, the `SAKE-PP` model jointly leverages the SAKE interaction mechanism (Eq. 11–14) and Laplacian Eigenvector Directional Aggregators to capture both local geometric relations (*via* positions and velocities) and global structural cues from the eigenvector-based directional GNN.

3.1 Cross-Validation Training and Model Generalization

To examine the impact of hierarchical iRMSD-guided sampling on model learning, we compared SAKE-PP trained on the hierarchically sampled decoy set with a no-sampling baseline (SAKE-PP-ns) trained on the corresponding unsampled top-100 ZDock-ranked decoys per complex. Such an analysis is intended to assess whether the sampling strategy provides a more informative learning regime for capturing structure–quality relationships among docking decoys. Under these respective data-construction protocols, the sampled setting yielded substantially lower regression error and higher correlation across five-fold cross-validation (Table S1), with MAE changing from 3.45 ± 0.01 to 0.95 ± 0.02, RMSE from 4.34 ± 0.01 to 1.29 ± 0.03, and Pearson's R from 0.24 ± 0.03 to 0.62 ± 0.01. These results suggest that hierarchical iRMSD-guided sampling improves the learnability of decoy quality and leads to more consistent structure–quality ranking.

Table S1: Impact of Hierarchical iRMSD-Guided Sampling on Model Performance (mean ± Std)

**Model**	**MAE**	**RMSE**	**R (Corr.)**
Baseline (No Sampling)	3.4477 ± 0.0089	4.3445 ± 0.0145	0.2411 ± 0.0342
**Our Model** (Hierarchical Sampling)	**0.9451** ± 0.0157	**1.2912** ± 0.0275	**0.6164** ± 0.0118

Figure S2 summarizes the training dynamics of the None-strategy regime. The training loss curves (Figure S2a) show rapid initial convergence followed by a gradual plateau. The corresponding validation losses (Figure S2b) display moderate fold-to-fold variability but do not show evidence of systematic overfitting. Fold-wise test metrics (Figure S2c) indicate stable predictive performance across all five partitions. The learning-rate schedules (Figure S2d) further show that step-decays occur at fold-specific epochs, reflecting heterogeneous convergence behavior across data splits.

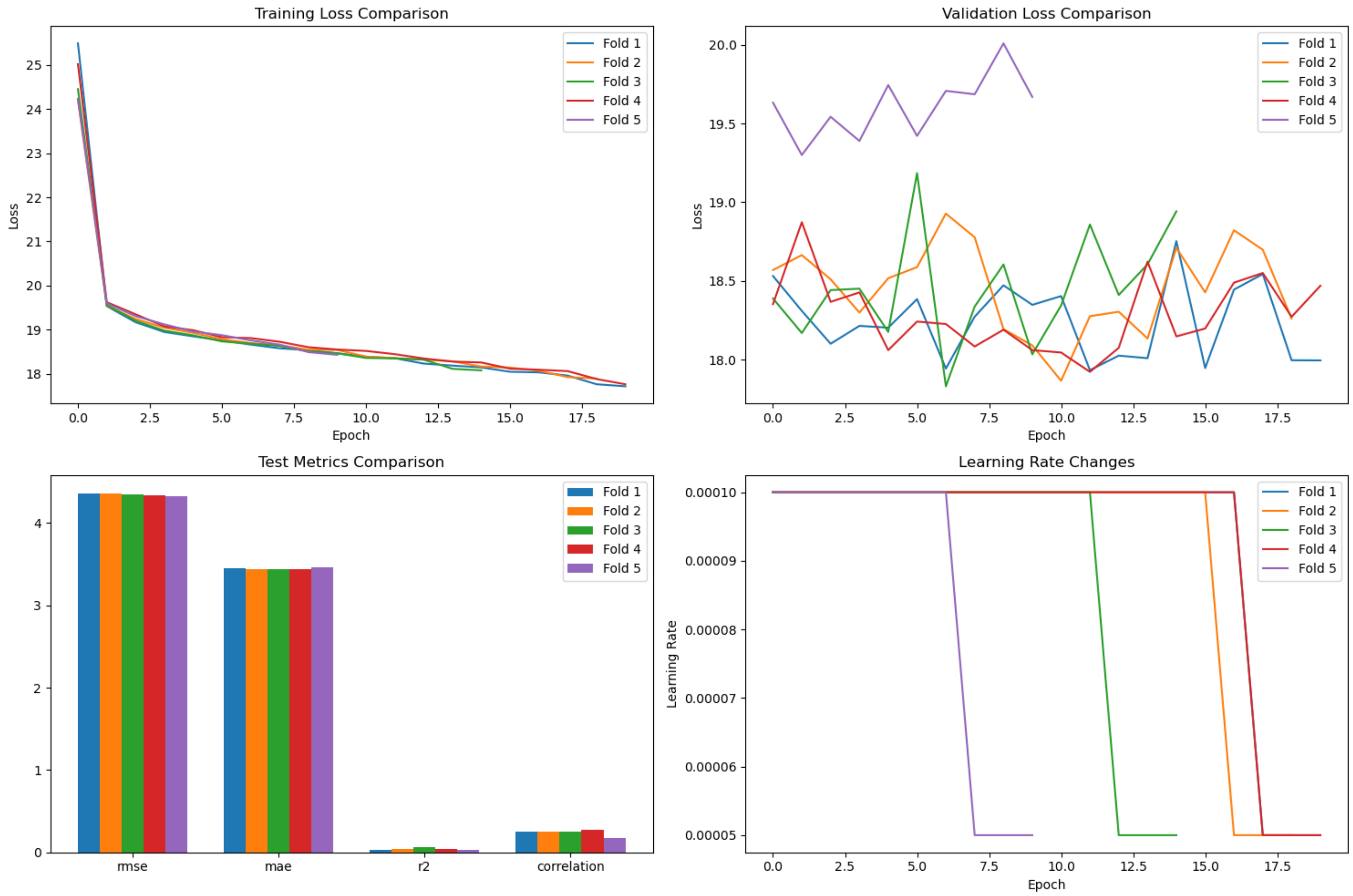

Figure S2: Cross-validation training dynamics and performance evaluation under the None-strategy regime. (a) Epoch-wise training loss (MSE) for each of the five folds. (b) Validation loss trajectories for the same folds. (c) Fold-wise held-out performance measured by RMSE, MAE, coefficient of determination (R^2), and Pearson correlation. (d) Learning-rate schedules used during training. All folds start at 1.0×10^{-4} and undergo step-decays at fold-specific epochs.

Figure S3 presents the corresponding analysis for the Strategy regime. Relative to the None-strategy baseline, the Strategy protocol extends training to 30 epochs and introduces two successive learning-rate reductions (from 1.0×10^{-4} to 5.0×10^{-5} and then to 2.5×10^{-5}) at fold-adaptive breakpoints (Figure S3d). This adaptive schedule leads to smoother validation trajectories (Figure S3b) and more stable convergence near 1.6 by epoch 30. Fold-wise held-out metrics (Figure S3c) are comparable to or better than those of the None-strategy regime, with reduced inter-fold variance in R^2 and Pearson correlation.

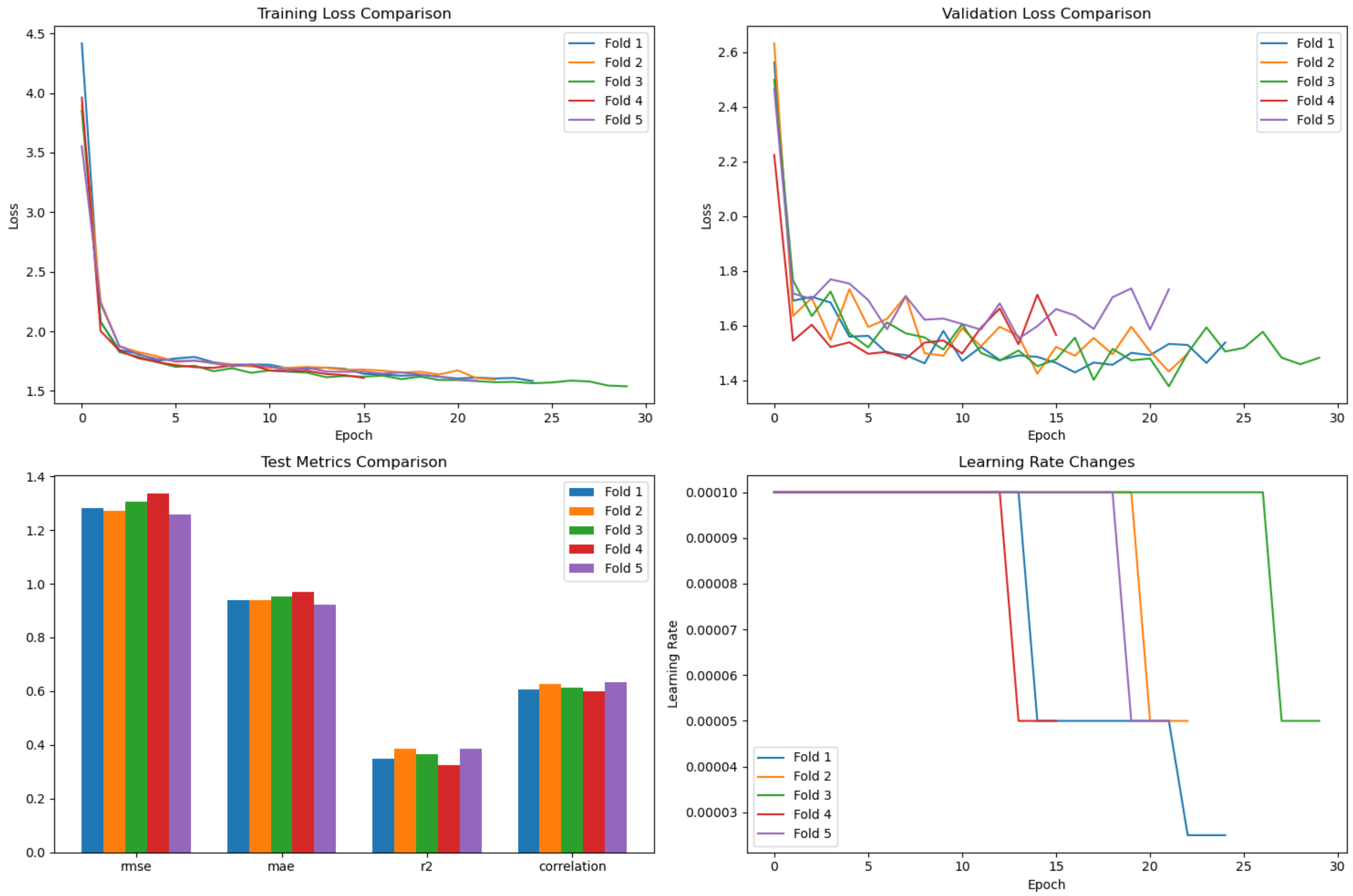

Figure S3: Cross-validation training dynamics and performance under the Strategy regime. (a) Epoch-wise training loss (MSE) for the five folds. (b) Validation loss trajectories across folds. (c) Fold-wise held-out performance measured by RMSE, MAE, coefficient of determination (R^2), and Pearson correlation. (d) Learning-rate schedules with two fold-adaptive step-decays from 1.0×10^{-4} to 5.0×10^{-5} and then to 2.5×10^{-5}.

4 Molecular Dynamics Simulations

4.1 Molecular Dynamics Simulations of 8PE2 Decoy Complexes from Different Ranking Score

4.1.1 AF3 Ranking Score Selected 8PE2 MD Simulation Snapshots

To visualize the conformational evolution of the 8PE2 complex under the AF3 ranking score, we extracted structural snapshots at 10% intervals (10%–100%) from the 1 μs molecular dynamics trajectory. Each snapshot corresponds to a specific simulation timepoint, ranging from 100 ns (10%) to 1000 ns (100%). The reference structure is taken at 0% (t = 0 ns), and the Cα RMSD of Chain B (ligand, residues 443–563) is computed relative to this initial conformation using the Kabsch superimposition algorithm. The AF3 system

exhibits oscillatory RMSD behavior over the trajectory, with values ranging from 0.898 Å (20%, 200 ns) to 1.810 Å (60%, 600 ns), and a mean Cα RMSD of 1.43 ± 0.28 Å across all non-zero timepoints. A notable conformational excursion is observed at 60%–70% of the trajectory, after which the structure partially recovers. The following figures present the structural snapshots at each interval, with the corresponding RMSD values annotated in the captions.

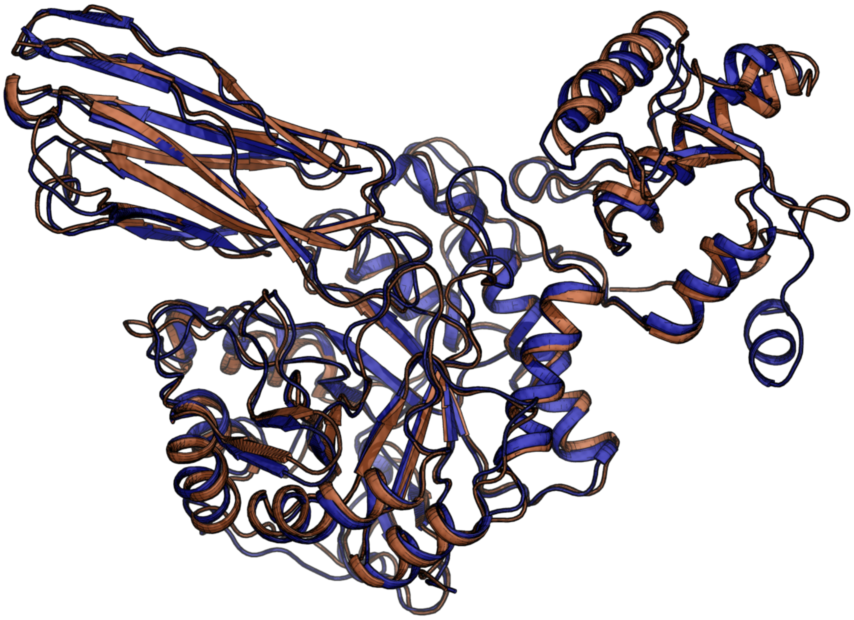

Figure S4: AF3 ranking score predicted conformation at 10% of the MD trajectory (100 ns). The structure of the 8PE2 complex is shown with Chain B (ligand, residues 443–563) highlighted. Cα RMSD relative to the initial structure (0%): **1.565 Å**.

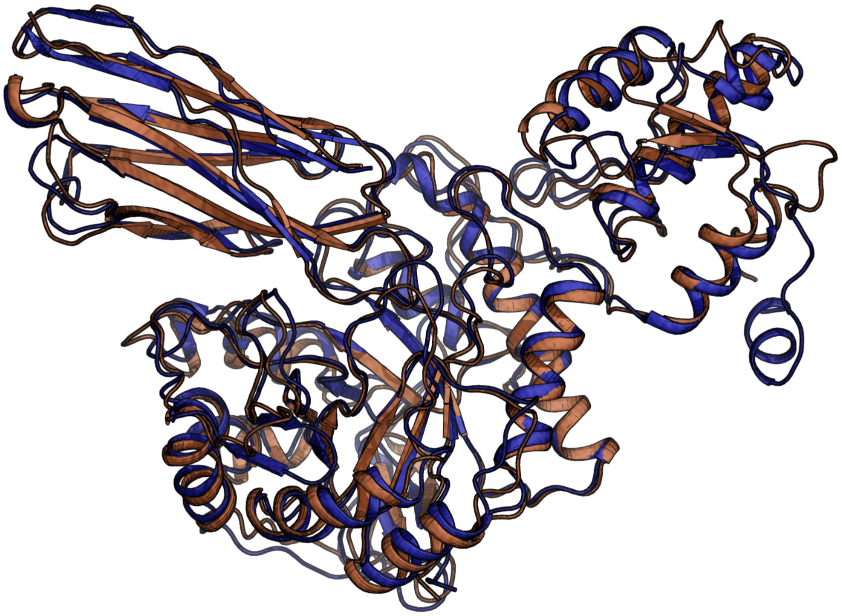

Figure S5: AF3 ranking score predicted conformation at 20% of the MD trajectory (200 ns). Cα RMSD vs initial: **0.898 Å**. This represents the minimum deviation observed across the entire trajectory, suggesting a transient return to near-initial conformation.

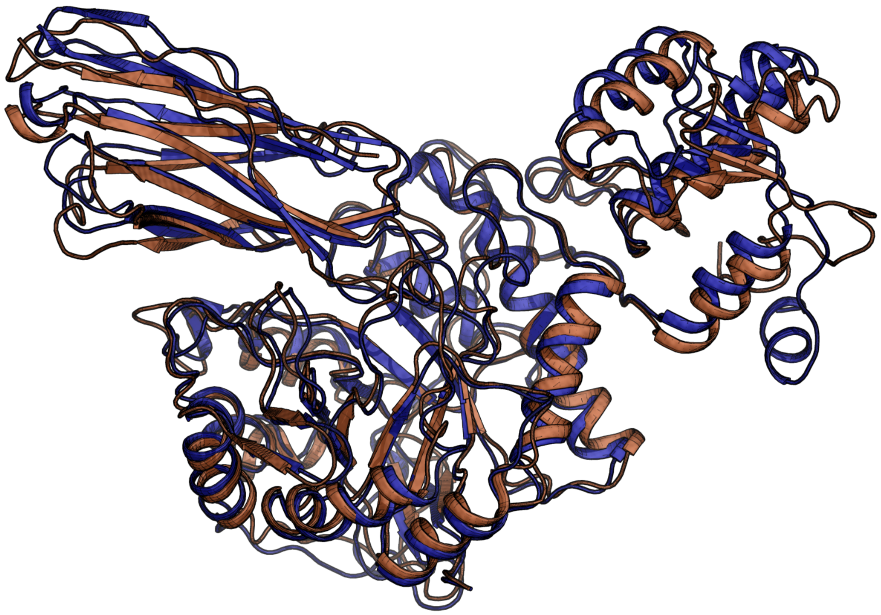

Figure S6: AF3 ranking score predicted conformation at 30% of the MD trajectory (300 ns). Cα RMSD vs initial: **1.435 Å**.

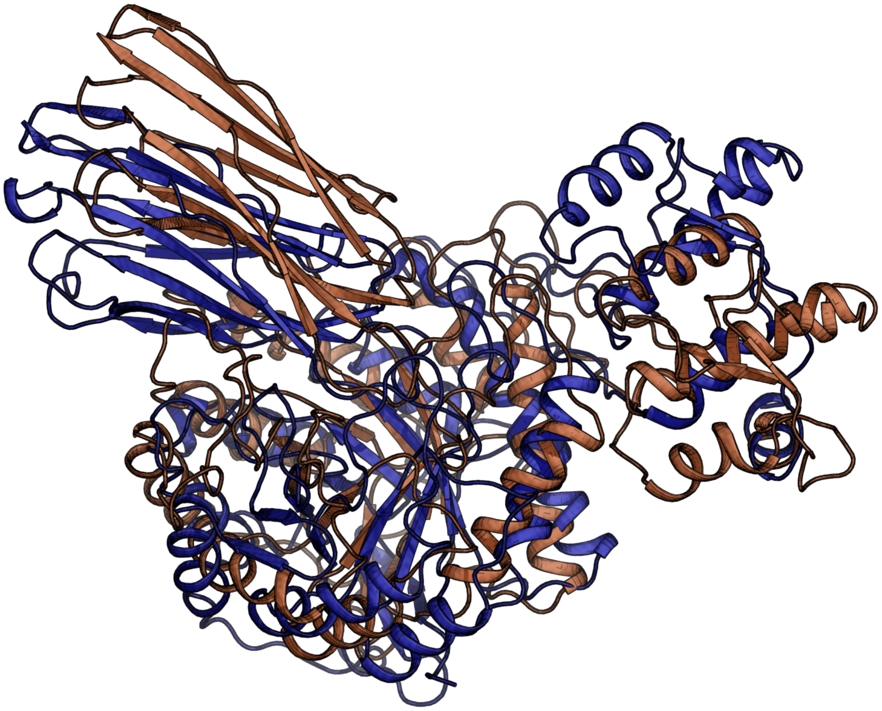

Figure S7: AF3 ranking score predicted conformation at 40% of the MD trajectory (400 ns). Cα RMSD vs initial: **1.036 Å**.

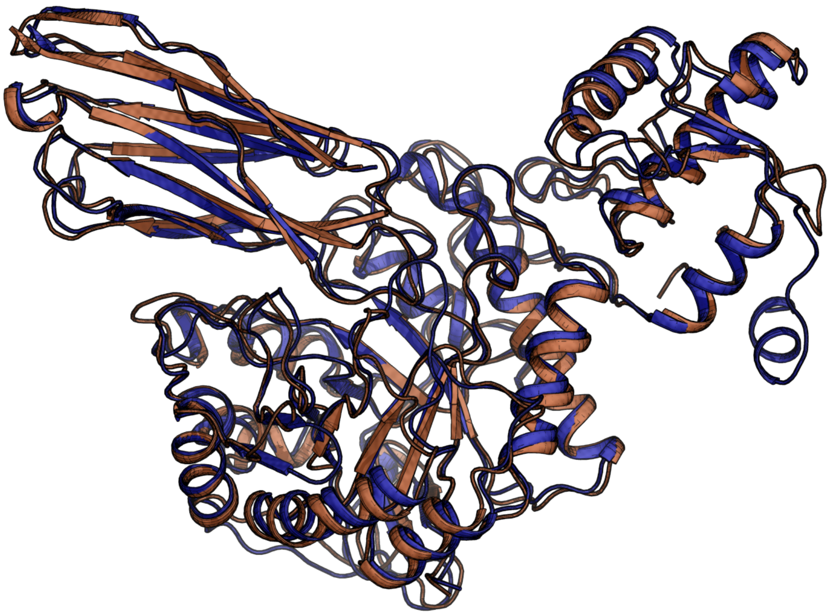

Figure S8: AF3 ranking score predicted conformation at 50% of the MD trajectory (500 ns). Cα RMSD vs initial: **1.310 Å**.

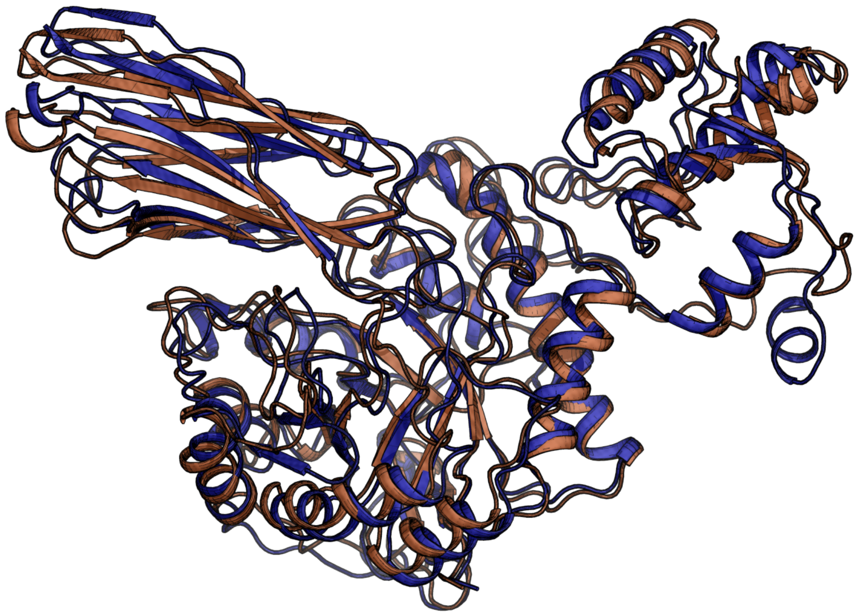

Figure S9: AF3 ranking score predicted conformation at 60% of the MD trajectory (600 ns). Cα RMSD vs initial: **1.810 Å**. This is the *maximum* deviation observed, indicating a transient conformational excursion of Chain B (ligand).

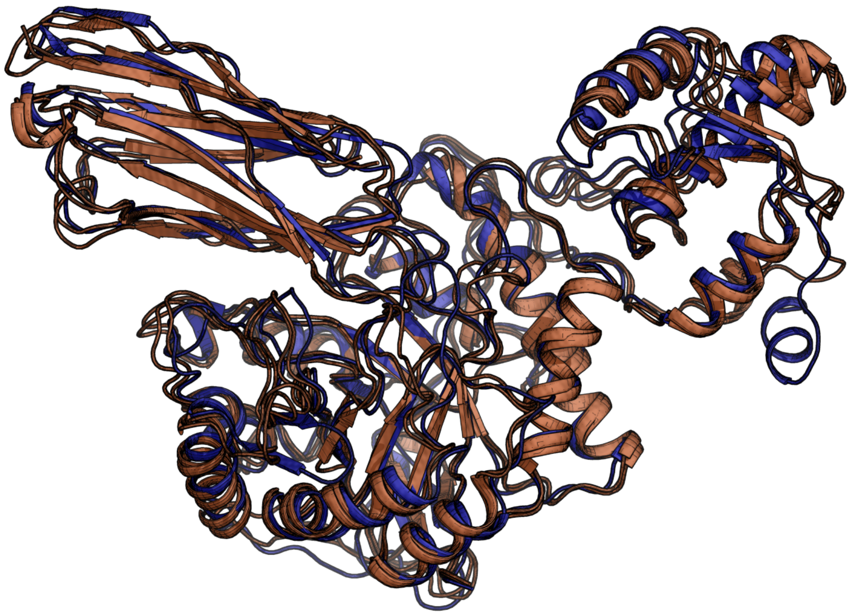

Figure S10: AF3 ranking score predicted conformation at 70% of the MD trajectory (700 ns). Cα RMSD vs initial: **1.701 Å**. The structure begins to recover from the peak deviation at 60%.

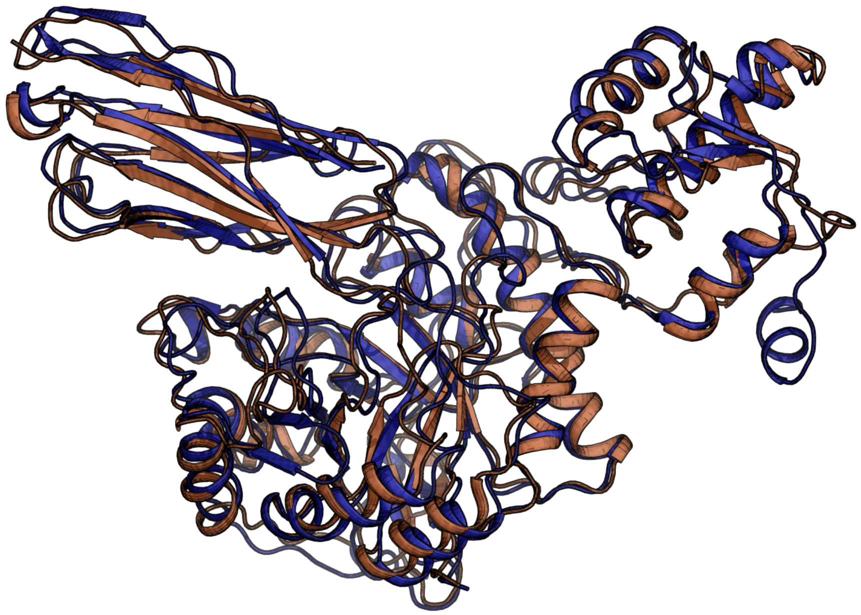

Figure S11: AF3 ranking score predicted conformation at 80% of the MD trajectory (800 ns). Cα RMSD vs initial: **1.511 Å**.

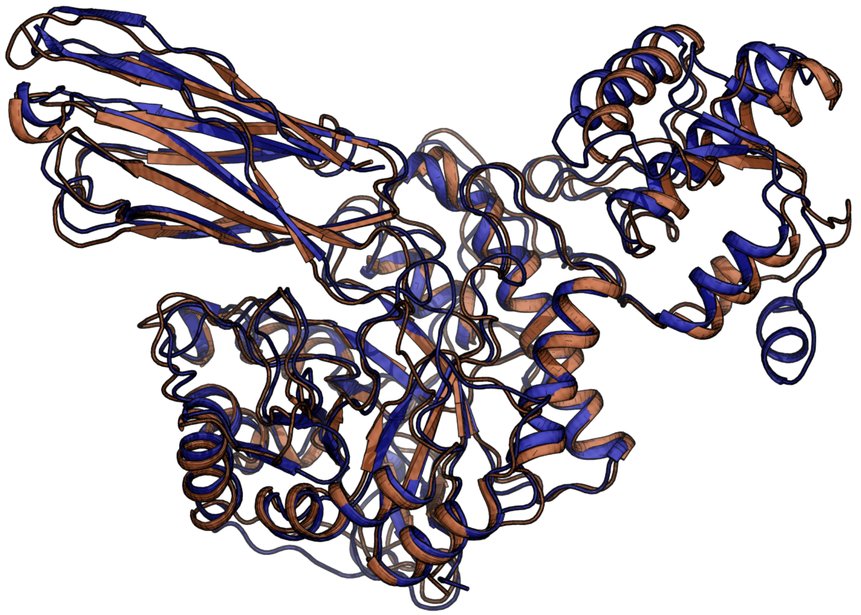

Figure S12: AF3 ranking score predicted conformation at 90% of the MD trajectory (900 ns). Cα RMSD vs initial: **1.589 Å**.

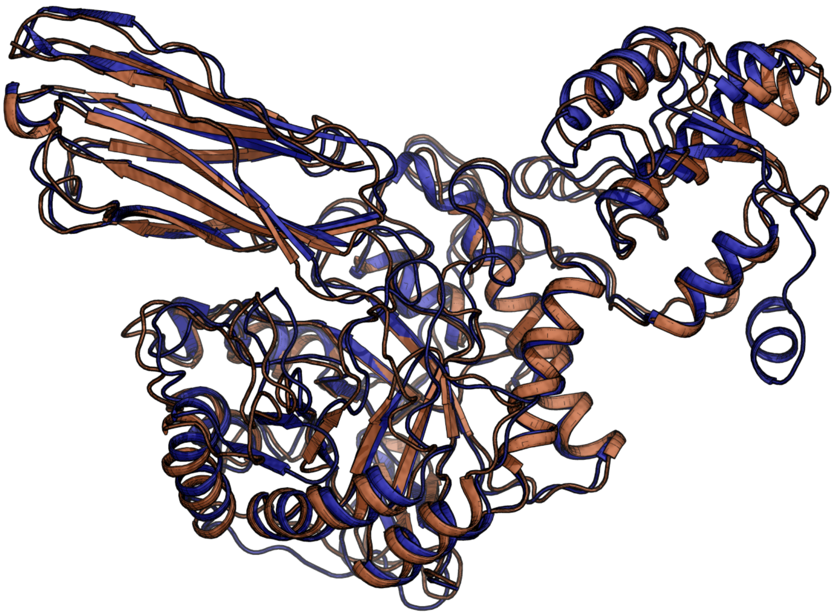

Figure S13: AF3 ranking score predicted conformation at 100% of the MD trajectory (1000 ns = 1 μs). Cα RMSD vs initial: **1.511 Å**. The final conformation remains within the stable fluctuation range (mean = 1.43 $\pm$ 0.28 Å), confirming no irreversible structural drift.

4.1.2 SAKE-PP Selected 8PE2 MD Simulation Snapshots

Analogous to the AF3 analysis above, we extracted structural snapshots at 10% intervals (10%–100%) from the 1 μs molecular dynamics trajectory of the 8PE2 complex generated using the SAKE-PP selected pose as the starting conformation. The Cα RMSD of Chain B (ligand, residues 443–563) was computed at each timepoint relative to the initial structure (0%, t = 0 ns) via the Kabsch superimposition algorithm. Compared to AF3, the SAKE-PP system demonstrates considerably more restrained conformational dynamics, with Cα RMSD values confined to the narrow range of 1.092 Å (80%, 800 ns) to 1.503 Å (30%, 300 ns) and a mean of 1.34 $\pm$ 0.15 Å—roughly half the standard deviation observed for AF3 ($\pm$ 0.28 Å). A distinctive two-phase behavior is evident: the first half of the trajectory (10%–60%) maintains a stable plateau around 1.4–1.5 Å, while the second half (70%–100%) exhibits a clear downward trend toward $\sim$1.1 Å, suggesting that the ligand progressively converges to a well-defined equilibrium binding pose. The following figures present the structural snapshots at each interval, with the corresponding RMSD values annotated in the captions.

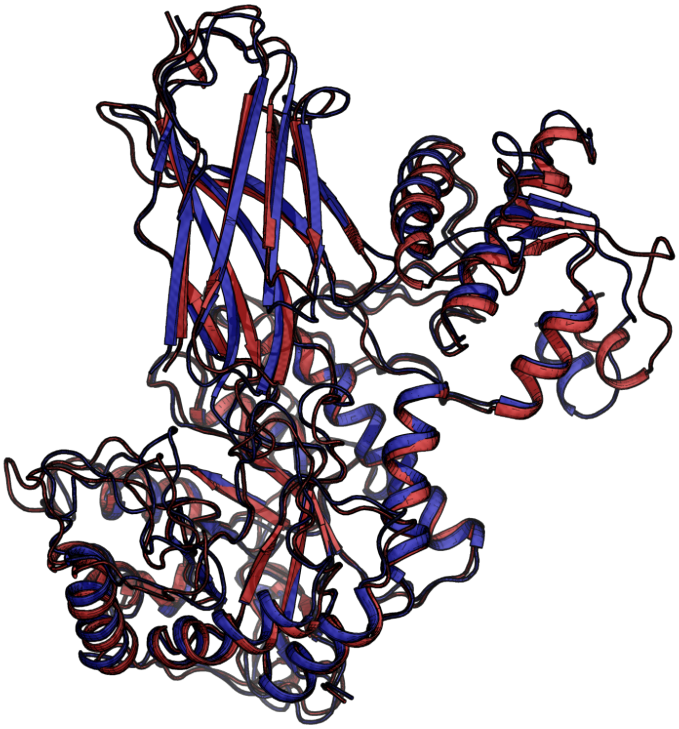

Figure S14: SAKE-PP score predicted conformation at 10% of the MD trajectory (100 ns). The structure of the 8PE2 complex is shown with Chain B (ligand, residues 443–563) highlighted. Cα RMSD relative to the initial structure (0%): **1.442 Å**.

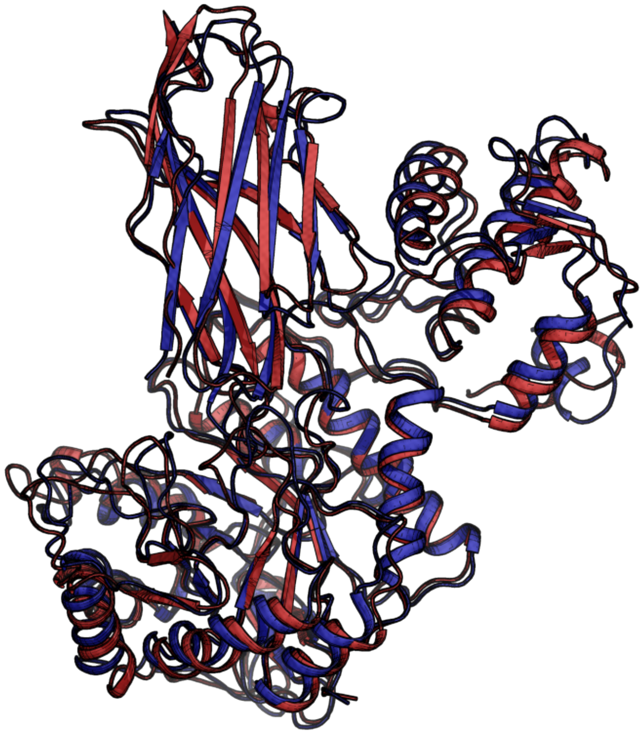

Figure S15: SAKE-PP score predicted conformation at 20% of the MD trajectory (200 ns). Cα RMSD vs initial: **1.455 Å**.

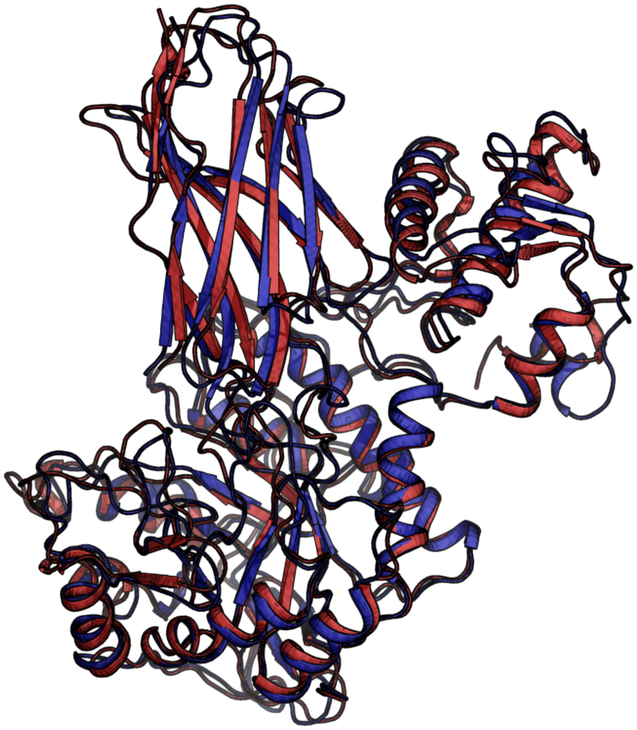

Figure S16: SAKE-PP score predicted conformation at 30% of the MD trajectory (300 ns). Cα RMSD vs initial: **1.503 Å**. This represents the *maximum* deviation observed for SAKE-PP, which is still notably lower than the AF3 peak (1.810 Å).

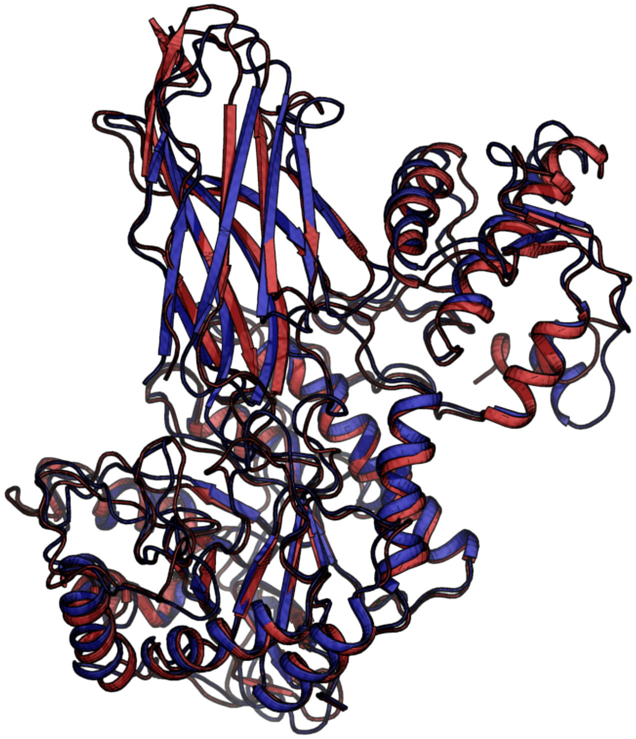

Figure S17: SAKE-PP score predicted conformation at 40% of the MD trajectory (400 ns). Cα RMSD vs initial: **1.398 Å**.

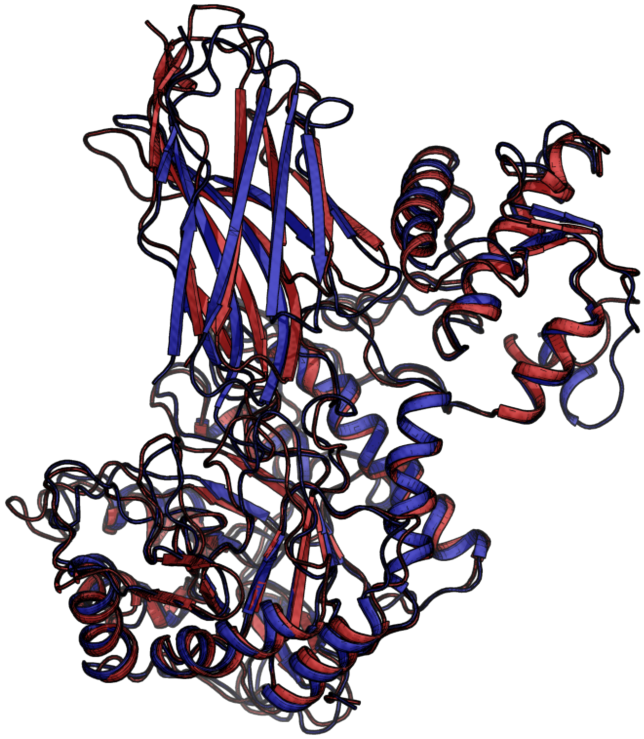

Figure S18: SAKE-PP score predicted conformation at 50% of the MD trajectory (500 ns). Cα RMSD vs initial: **1.429 Å**.

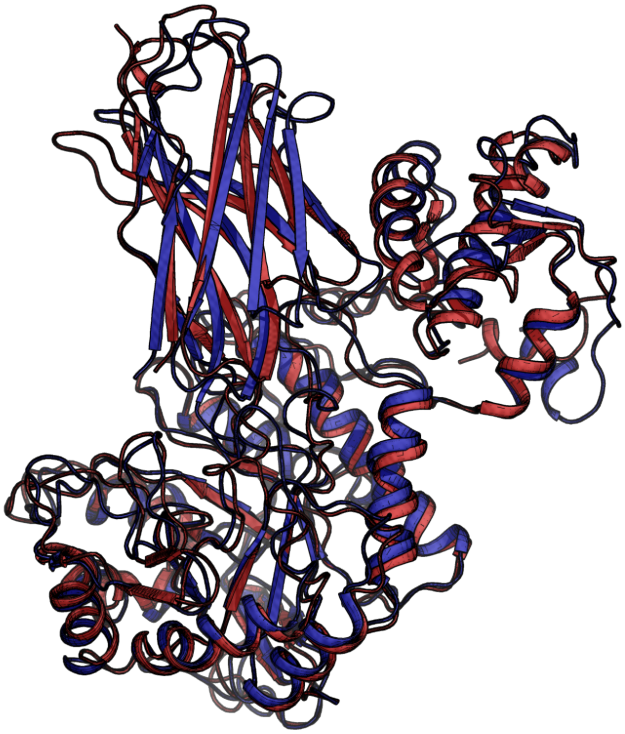

Figure S19: SAKE-PP score predicted conformation at 60% of the MD trajectory (600 ns). Cα RMSD vs initial: **1.454 Å**. At this timepoint, AF3 reaches its peak deviation of 1.810 Å, while SAKE-PP remains stable.

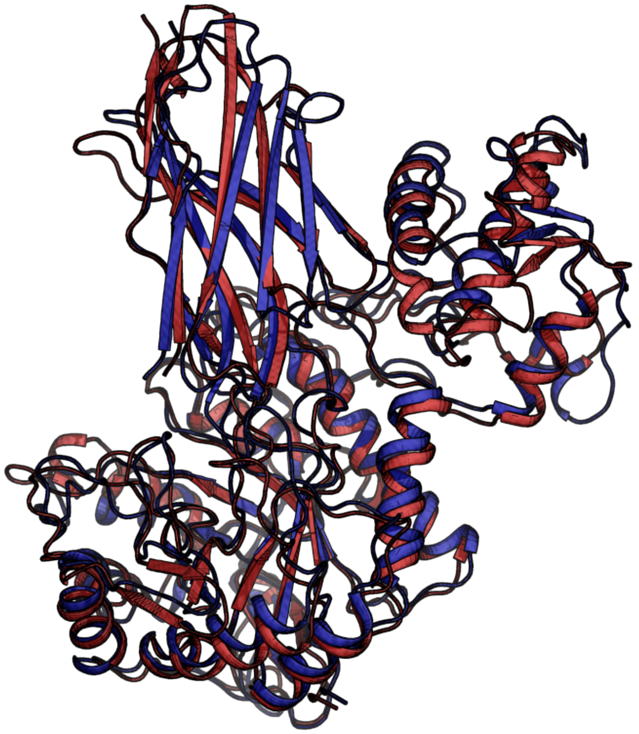

Figure S20: SAKE-PP score predicted conformation at 70% of the MD trajectory (700 ns). Cα RMSD vs initial: **1.130 Å**. A significant decrease from the first-half plateau, marking the onset of convergence.

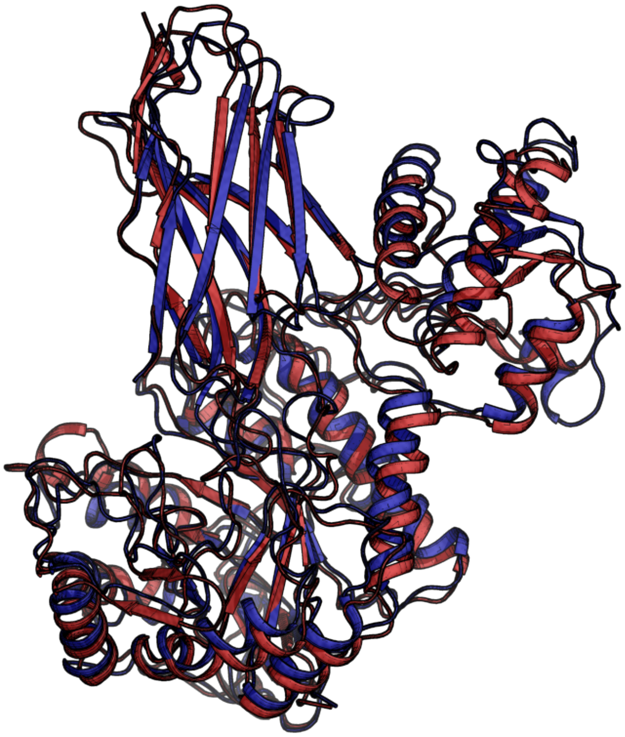

Figure S21: SAKE-PP score predicted conformation at 80% of the MD trajectory (800 ns). Cα RMSD vs initial: **1.092 Å**. This is the *minimum* deviation observed, indicating the ligand has reached its most stable conformation.

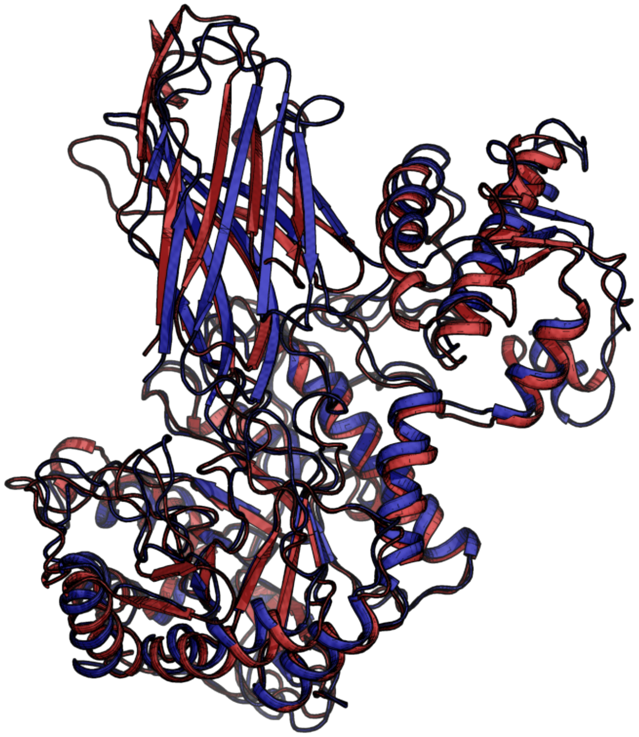

Figure S22: SAKE-PP score predicted conformation at 90% of the MD trajectory (900 ns). Cα RMSD vs initial: **1.192 Å**.

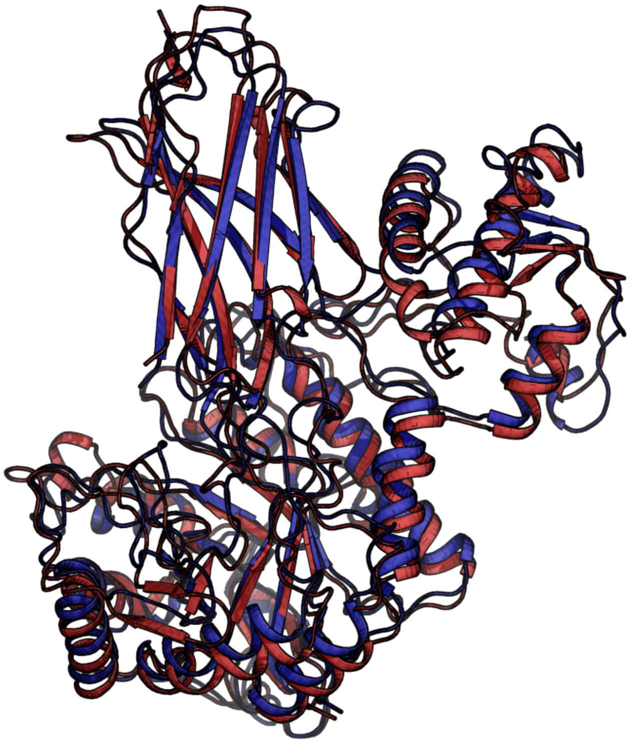

Figure S23: SAKE-PP score predicted conformation at 100% of the MD trajectory (1000 ns = 1 μs). Cα RMSD vs initial: **1.273 Å**. The final conformation remains well within the stable range (mean = 1.34 ± 0.15 Å), confirming sustained structural stability of the ligand throughout the entire simulation.

4.1.3 Comparative Cα RMSD Analysis of AF3 and SAKE-PP Systems

Having examined the individual conformational trajectories of the AF3 and SAKE-PP systems in the preceding sections, we now present a direct comparative analysis to quantitatively assess the relative structural stability of the two docking-derived starting poses over the course of the 1 μs MD simulation. All RMSD values reported below refer to Cα atoms of Chain B (ligand, residues 443–563), computed relative to the respective initial structure (0%, $t = 0$ ns) via the Kabsch superimposition algorithm.

Figure S24 overlays the Cα RMSD drift profiles of both systems on a common time axis. The AF3 system (blue) exhibits pronounced oscillatory behavior with RMSD values spanning from 0.898 Å (200 ns) to 1.810 Å (600 ns), yielding a mean of 1.43 ± 0.28 Å and a coefficient of variation (CV) of approximately 19.6%. In contrast, the SAKE-PP system (red) displays markedly reduced fluctuations, confined to the narrow range of 1.092 Å (800 ns) to 1.503 Å (300 ns), with a mean of 1.34 ± 0.15 Å and a CV of $\sim$11.2%. Two key

observations emerge: (i) the SAKE-PP trajectory demonstrates a clear convergence trend in the second half of the simulation (700–1000 ns), where RMSD values systematically decrease toward ∼1.1–1.3 Å, indicative of the ligand settling into a well-defined equilibrium binding pose; and (ii) the shaded ΔRMSD region between the two curves highlights timepoints at which the AF3 system undergoes conformational excursions not mirrored by SAKE-PP, most prominently at 600–700 ns. The substantially lower standard deviation of the SAKE-PP system (± 0.15 Å vs. ± 0.28 Å for AF3) suggests that the SAKE-PP–selected starting conformation resides closer to a thermodynamic energy minimum, thereby requiring less conformational rearrangement to achieve equilibrium.

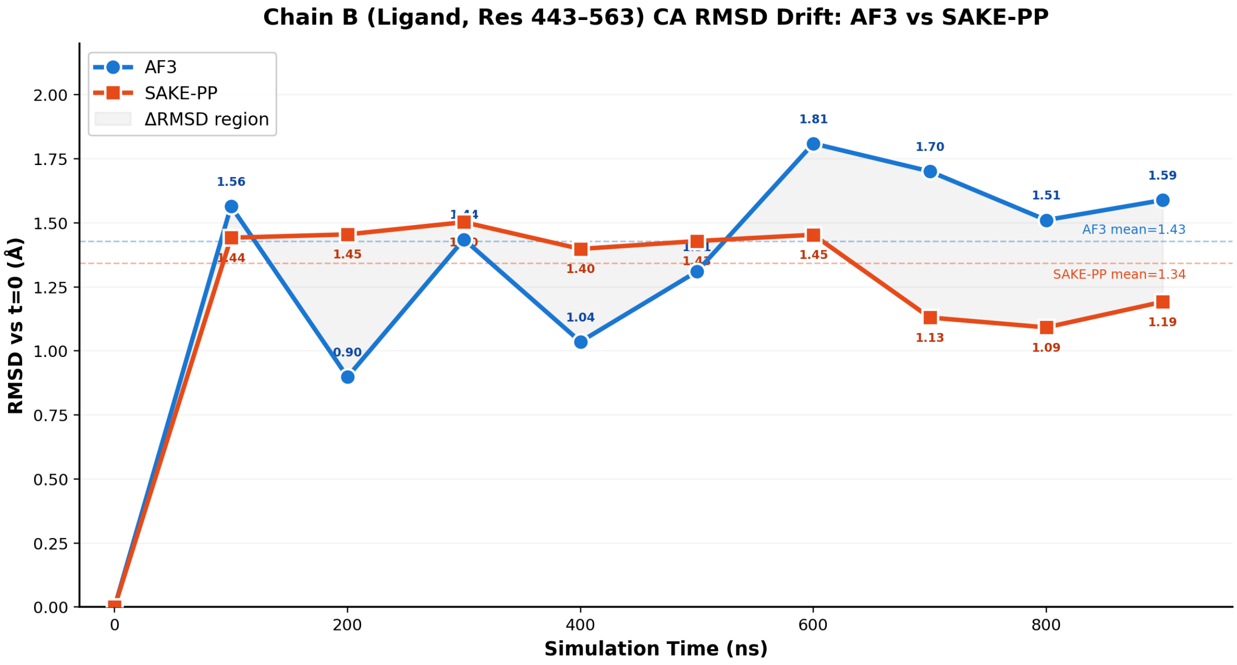

Figure S24: Comparative Cα RMSD drift of Chain B (ligand, residues 443–563) for the AF3 (blue) and SAKE-PP (red) systems over the 1 μs MD trajectory. RMSD is computed relative to the respective initial structure (0%). Dashed horizontal lines indicate the mean RMSD for each system (AF3: 1.43 ± 0.28 Å; SAKE-PP: 1.34 ± 0.15 Å). The shaded region represents the ΔRMSD envelope between the two profiles.

To confirm that the observed stability trends are not an artifact of the Cα-only atom selection, we extended the analysis to two additional atom sets: backbone atoms (N, Cα, C, O; 484 atoms per frame) and all heavy (non-hydrogen) atoms (943 atoms per frame). Figure S25 presents the RMSD drift profiles for all three metrics side by side. Across all atom selections, the SAKE-PP system consistently exhibits lower mean RMSD and reduced variance relative to AF3. The backbone RMSD trends closely parallel those of Cα, while the heavy-atom RMSD—which captures side-chain dynamics in addition to backbone fluctuations—shows a modest upward shift for both systems but preserves the same relative ordering. This consistency across hierarchical structural descriptors strongly indicates that the enhanced stability of the SAKE-PP complex

is a genuine property of the ligand–receptor binding mode, rather than a selective feature of Cα geometry alone.

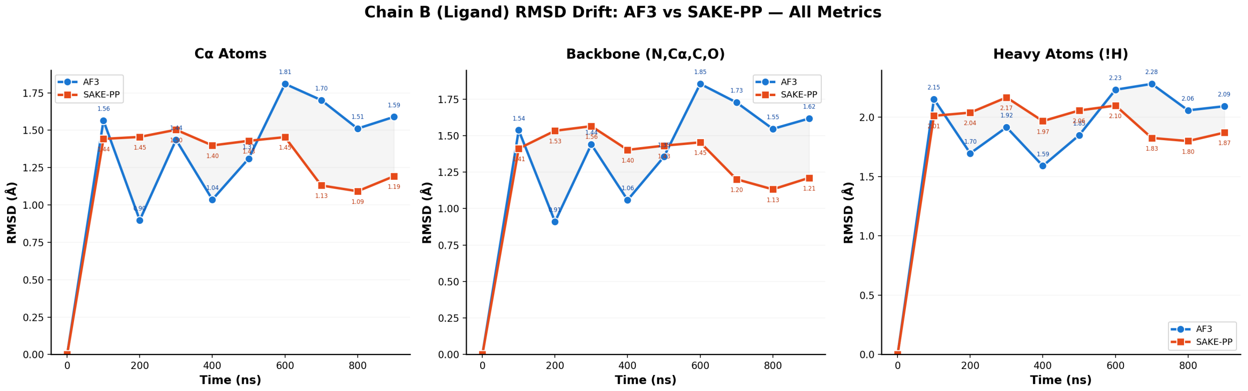

Figure S25: RMSD drift of Chain B (ligand) for the AF3 and SAKE-PP systems computed using three atom selections: Cα atoms (left), backbone N/Cα/C/O atoms (center), and all heavy (non-hydrogen) atoms (right). The shaded region in each panel indicates the ΔRMSD envelope. The SAKE-PP system shows consistently lower mean RMSD and variance across all metrics.

To further elucidate the timepoint-resolved differences, we computed the signed Cα RMSD difference ($\Delta\text{RMSD} = \text{RMSD}_{\text{AF3}} - \text{RMSD}_{\text{SAKE-PP}}$) at each 10% interval, as depicted in Figure S26. Positive values (red) denote timepoints where AF3 exhibits greater structural deviation than SAKE-PP, while negative values (green) indicate the converse. During the first 500 ns (10%–50%), the difference oscillates around zero, with AF3 transiently showing lower RMSD at 200 ns ($\Delta = -0.557$ Å) and 400 ns ($\Delta = -0.362$ Å). However, from 600 ns onward, ΔRMSD becomes consistently and substantially positive, peaking at $+0.571$ Å (700 ns) and remaining above $+0.35$ Å through the end of the simulation. This late-trajectory divergence coincides with the convergence phase of SAKE-PP and the sustained conformational excursion of AF3, reinforcing the interpretation that SAKE-PP achieves a more thermodynamically favorable and kinetically stable binding configuration.

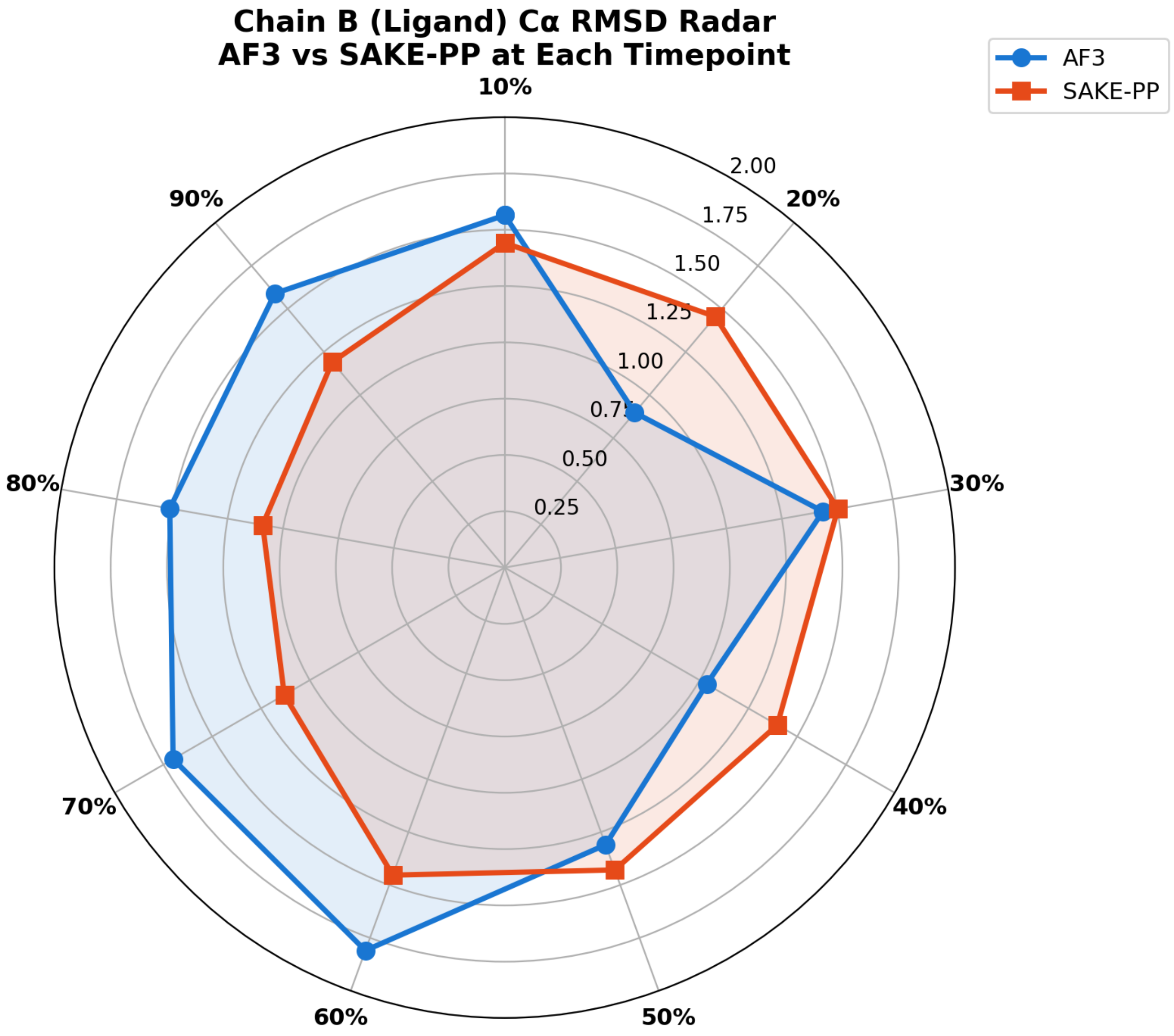

Figure S26: Radar plot comparing the Cα RMSD of Chain B (ligand, residues 443–563) between the AF3 (blue) and SAKE-PP (red) systems at each 10% trajectory interval. Each axis corresponds to a simulation timepoint (10%–90%). The smaller enclosed area of the SAKE-PP profile reflects its consistently lower and more uniform RMSD values across all timepoints.

Taken together, these analyses demonstrate that both the AF3- and SAKE-PP–derived complexes maintain overall structural integrity over the full 1 μs simulation—neither system undergoes irreversible unfolding or ligand dissociation. However, the SAKE-PP system exhibits quantitatively superior stability by every metric considered: lower mean RMSD (1.34 vs. 1.43 Å), smaller fluctuation amplitude ($\pm$ 0.15 vs. $\pm$ 0.28 Å), and a clear late-trajectory convergence toward an equilibrium binding pose. From a thermodynamic perspective, the reduced conformational entropy implied by the narrower RMSD distribution of SAKE-PP is consistent with a tighter, more enthalpically favorable ligand–receptor interface, suggesting that the SAKE-PP scoring function identifies starting conformations that are closer to the global free-energy minimum of

the bound complex.

4.2 8K0E, 8YF2, 8S4K, 8SOZ, 8VGG MD Simulation detail

To provide a direct structural comparison between the two scoring methods, Figure S27 summarizes the decoy-selection outcomes for three representative targets (8XYZ, 8YF2, and 8K0E).

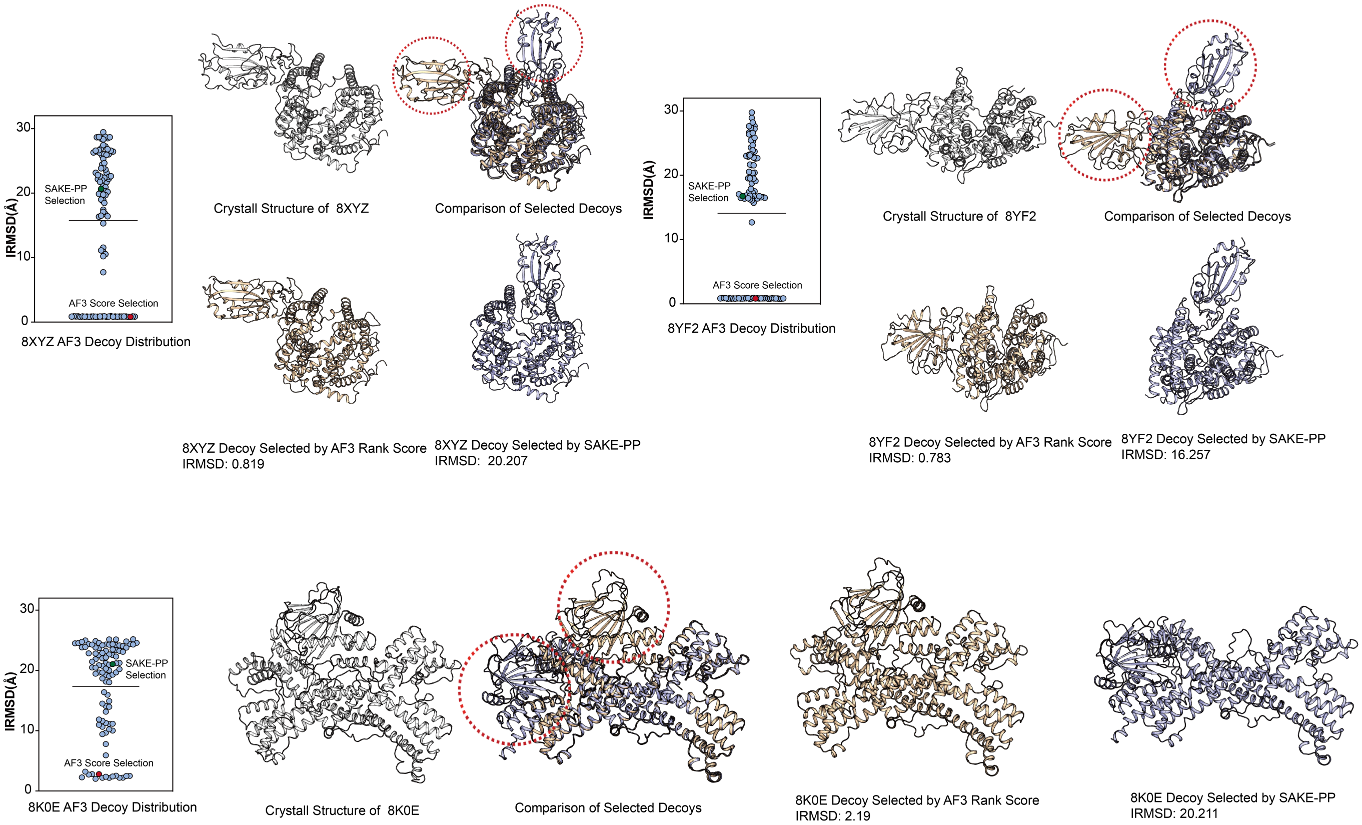

Figure S27: (a, d, k) Scatterplots of iRMSD for all 100 AlphaFold3 (AF3)–generated decoys of PDB IDs 8XYZ, 8YF2, and 8K0E, respectively. The green marker indicates the decoy chosen by SAKE-PP, while the red marker denotes the one selected by the AF3 ranking score. The grey horizontal line marks the iRMSD of the SAKE-PP selection. Percentages report the fraction of decoys whose iRMSD is equal to or lower than that of the AF3-selected model. (b, e, l) Crystal structures of the three targets, rendered in grey. (c, f, m) Structural overlays of the two selected decoys: the AF3 choice is shown in wheat, the SAKE-PP choice in blue. Red dashed circles highlight interface regions where the two selections diverge markedly. (g, i, n) Isolated views of the decoys selected by AF3, with their corresponding iRMSD values. (h, j, o) Isolated views of the decoys selected by SAKE-PP, also annotated with their iRMSD values.

The complexes of 8K0E, 8YF2, 8S4K, 8SOZ, 8VGG, and MOD were predicted by both AF3 ranking score and SAKE-PP. All complexes were prepared using the leap module in AMBER20 package. All MD simulations were performed using pmemd.cuda in AMBER20 with ff14SB force field for the protein. All simulations were carried out in a truncated octahedron box formed by TIP3P explicit water molecules. The

closest distance between any atom originally present in solute and the edge of the periodic box is 12 Å. The particle mesh Ewald (PME) method is used to treat the long-range electrostatic interactions. The nonbonded interactions are truncated with 10 Åcutoff. Periodic boundary condition (PBC) is imposed on the system during the calculation of nonbonded interactions. The time-step is set at 2 fs and SHAKE is used to constrain the bonds involving hydrogen atoms. Langevin thermostat with the collision frequency 2.0 is applied to control the temperature.

First, the system is minimized with protein constrained to equilibrate the solvent. Then, the protein is released to minimize the whole simulation system. The system is slowly heated to 300 K, followed by a 40 ns equilibration of the whole system in an NPT ensemble at an interval of every 10 fs, and during this section, no restraint is performed. Then a 10 ns simulation was performed to obtain 5000 snapshots for ASGBIE analysis.

Table S2: Predicted protein–protein binding free energies (kcal/mol) by AF3 and SAKE.

PDB ID	AF3 ranking score	SAKE-PP
8K0E	-71.41	-75.77
8YF2	-26.39	-50.99
8S4K	-35.25	-62.09
8SOZ	-2.84	-16.98
8VGG	-46.01	-53.88
8XYZ	-29.39	-30.97

For the six heterogeneous case studies discussed in the main text, we performed corresponding molecular dynamics simulations, and the results consistently demonstrate that SAKE-PP exhibits superior binding energies.

In the 8VGG–SAKE-PP selected decoy(Figure S28), chain B contains four disulfide bonds, whereas in the 8VGG–AF3 ranking score decoy only three are formed. As a result, the folded segment comprising residues 187–206 in chain B is displaced away from the rest of the complex. Although this folded region does not directly contact the partner protein, the presence of the additional disulfide bond markedly stabilizes the local fold and thus enhances the stability of the binding interface. In particular, the absence of the S–S linkage between Cys 140 and Cys 191 in the AF3 prediction weakens the binding contributions of residues Ser 122, Asp 123, Phe 119, Pro 120, Val 134, Leu 136, Cys 125, Gln 161, Thr 165, Ser 175, and Thr 181 by approximately 0.5 kcal/mol compared to SAKE-PP. Notably, the disulfide bond at Thr 181 in chain B increases the free-energy contribution of that residue by 1.24 kcal/mol. Moreover, these residues are predominantly polar, indicating that a more rigid scaffold promotes the formation of stabilizing hydrogen bonds.

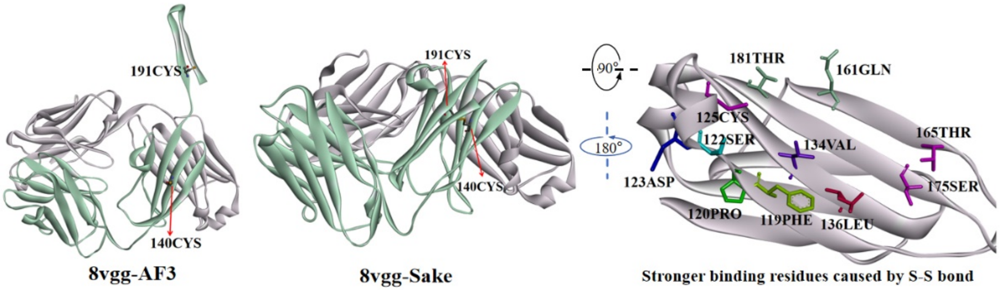

Figure S28: Differences in disulfide-bond connectivity in the 8VGG system as predicted by AF3 and SAKE-PP, and key residues with significant binding-energy contributions in the SAKE-PP model.

In the 8YF2-SAKE-PP system, there were 86 interface residues, compared to 59 in 8YF2-AF3 ranking score, resulting in an additional 27 residues (Figure S2) contributing -9.21 kcal/mol to the binding energy. Notably, residue 17TYR contributed 4.44 kcal/mol, while 12ARG contributed 3.38 kcal/mol. Positively charged residues such as 175ARG, 90LYS, 132ARG, and 21ARG all contributed over 2 kcal/mol, while the polar residue 136THR contributed 0.99 kcal/mol. These additional residues obtained by SAKE-PP provided new binding sites and regions for binding. Furthermore, among the interface residues common to both

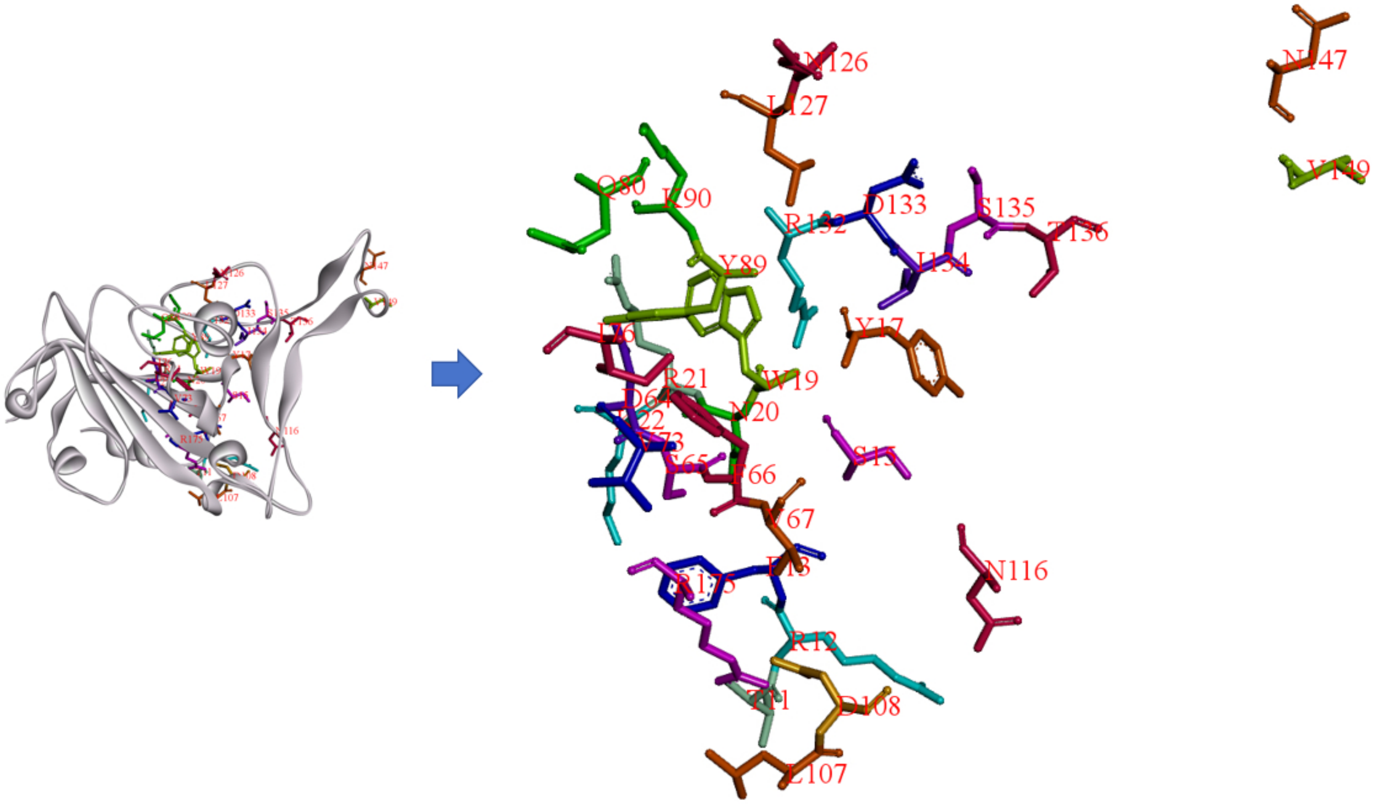

Figure S29: Comparison of predicted interface hotspots in the 8YF2 complex using two scoring methods.

SAKE-PP and AF3 ranking score, residue 83LYS exhibited the largest increase, shifting from -2.36 kcal/mol

in AF3 ranking score to -7.05 kcal/mol in SAKE-PP. Residue 87TYR changed from a repulsive contribution of 0.21 kcal/mol in AF3 ranking score to a positive contribution to binding of -3.28 kcal/mol in SAKE-PP, indicating the contribution of the pi-system interactions. Additionally, residues 138ILE, 156PHE, and 159GLN showed increases in contribution exceeding 2 kcal/mol. Overall, the combined contributions of these residues in SAKE-PP not only introduced new binding sites but also enhanced the effects of overlapping residues (15.38 kcal/mol), with electrostatic interactions and pi-system interactions playing significant roles.

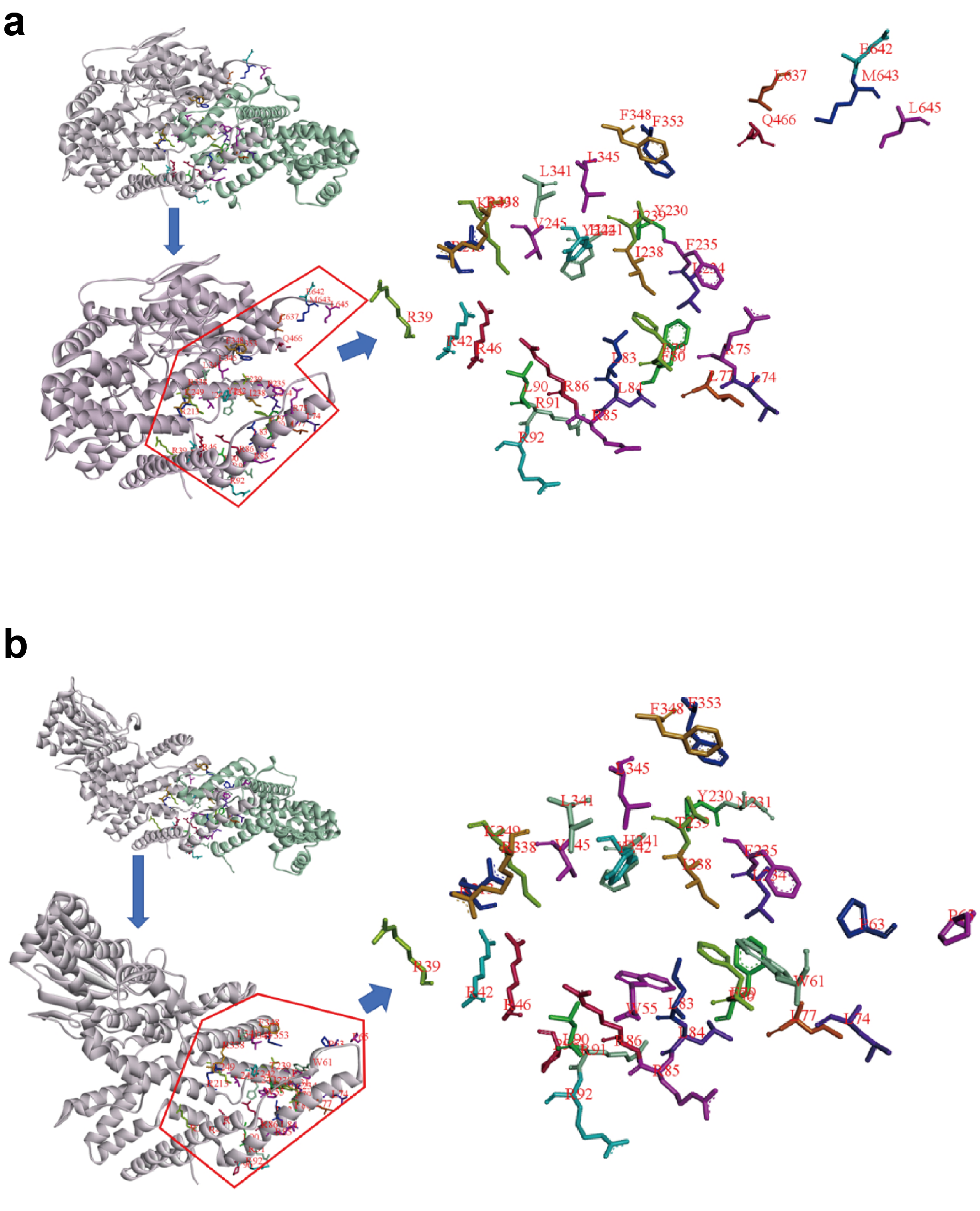

Figure S30: Comparison of predicted interface hotspots in the 8K0E complex using two scoring methods: (a) hotspots identified by the AF3 ranking score; (b) hotspots identified by the SAKE-PP score.

In 8S4K-AF3, the loop connecting region between the two β-systems is designed as a straight chain (Figure S31). The major difference from the conformation obtained by SAKE-PP lies in straightening out two helical regions and two Ω-loops, resulting in a significant deviation of the β-stacking region near the C-terminus from the corresponding region of the other protein molecule, leading to a significant weakening of their interaction. For 8S4K-SAKE-PP, there are 110 interface residues, while 8S4K-AF3 has 67, resulting in an additional contribution of 43 residues to the binding energy of $-21.85\,\text{kcal/mol}$, including 13 new hot spot residues. Especially, residues such as 160^{PHE}, 137^{LYS}, 135^{LEU}, 158^{HIE}, 163^{VAL}, 174^{SER}, 165^{GLN}, 174^{SER}, and 165^{GLN} show an increase of 1 kcal/mol in binding energy compared to AF3. Additionally, among the shared hot spot residues predicted in the two structures, 125^{LEU} contributes an increase of 2.70 kcal/mol to binding, 32^{GLN} increases by 1.63 kcal/mol, 131^{THR} by 1.56 kcal/mol, and 123^{TYR} by 0.86 kcal/mol. Therefore, 8S4K-SAKE-PP not only adds to the binding region compared to 8S4K-AF3 but also enhances the residue interactions in the original overlapping region by 4.58 kcal/mol.

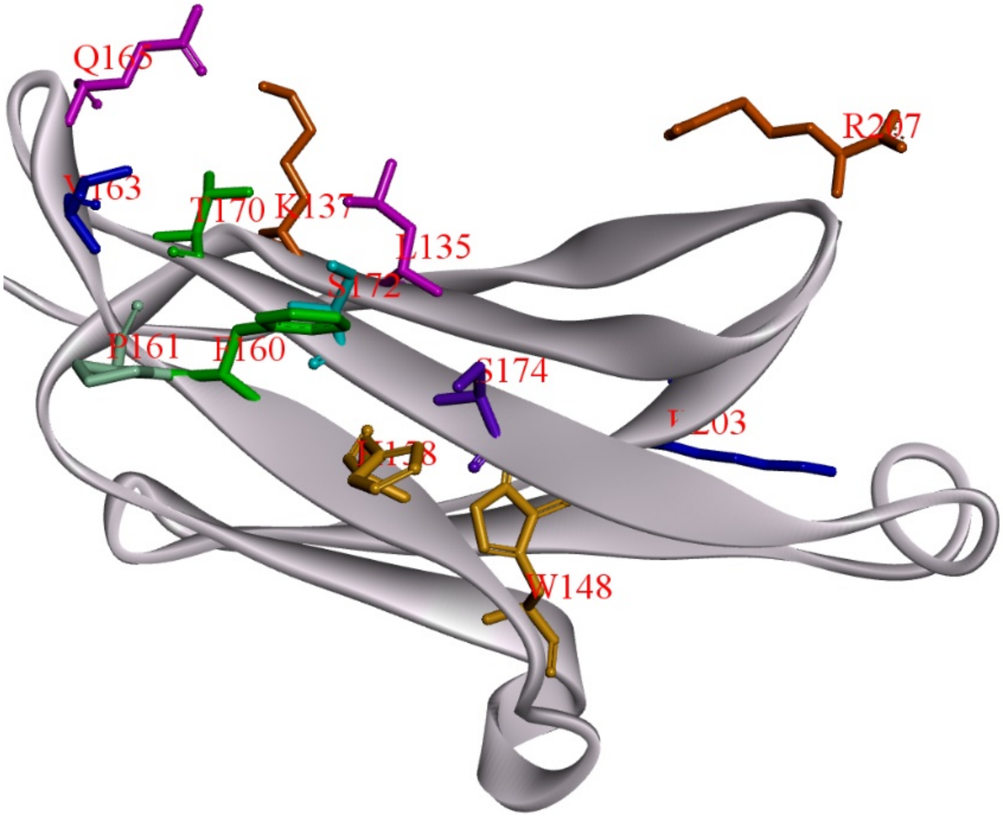

Figure S31: Additional hotspot residues identified by SAKE-PP compared to AF3 in the 8S4K system.

In the 8XYZ system, both SAKE-PP and AF3 ranking score selections yield complexes with highly similar RBD–Spike protein architectures. The AF3 ranking score selection results show a binding pattern similar to the traditional WT RBD and mutant RBD binding to the ACE2 region, whereas the results

obtained by SAKE enable RBD to explore a new binding mode. By binding in this new mode, SAKE-PP identifies new binding sites with a total of 30 residues contributing 2.66 kcal/mol to the binding free energy. While the contribution of overlapping residues with AF3 is slightly reduced (1.08), the additional binding sites compensate for the energy deficiency.

Therefore, considering the predictions from AF3 ranking score and SAKE-PP, AF3 identifies more binding sites in 8K0E, with the additional sites contributing favorably to binding by 1.78 kcal/mol. However, the contribution to overlapping sites is significantly weakened, indicating that the beneficial enhancement of the additional sites does not fully compensate for the weakening of other sites. In contrast, 8XYZ-SAKE-PP, with its additional binding sites, also leads to a decrease in the binding energy of the overlapping residues with AF3 ranking score, but the new region fully covers the decreased energy values. In other systems, while SAKE-PP acquires new binding sites, it also enhances the binding in the overlapping regions. Moreover, in predicting disulfide bonds, SAKE-PP compensates for the sites that decoy from AF3 Ranking Score failed to form, resulting in enhanced binding energy in that region, which not only confirms its predictive advantage in terms of energy but also demonstrates its capability in disulfide bond formation.

In the mod–SAKE-PP and mod–AF3 ranking score complexes, the overall architecture of the RBD–Spike interface is similar. The AF3 model reproduces the canonical binding mode of the wild-type and mutant RBDs to ACE2, whereas SAKE-PP uncovers an alternative binding pose. In this novel mode, SAKE-PP identifies 30 residues that collectively contribute 2.66 kcal/mol to the binding free energy. Although the overlapping residues with AF3 exhibit a slightly reduced per-residue contribution (–1.08 kcal/mol), the additional interactions compensate for this loss, yielding an overall more favorable interface.

Table S3: Per-residue $\Delta\Delta G$ contributions for 8K0E using AF3 ranking score vs. SAKE-PP.

**8K0E – AF3 ranking score**		**8K0E – SAKE-PP**	
**Residue**	$\Delta\Delta G$	**Residue**	$\Delta\Delta G$
242TYR	-6.71	91ARG	-6.53
91ARG	-6.41	242TYR	-6.05
86ARG	-6.22	92ARG	-5.41
77LEU	-5.31	77LEU	-5.05
84LEU	-4.83	84LEU	-4.95
234LEU	-4.12	235PHE	-4.73
353PHE	-4.10	86ARG	-4.01
235PHE	-3.68	83LEU	-4.00
238ILE	-3.52	353PHE	-3.79
241HIE	-3.38	234LEU	-3.78
74LEU	-3.11	241HIE	-3.67
345LEU	-3.03	345LEU	-3.42
341LEU	-3.01	238ILE	-3.10
338ARG	-2.84	74LEU	-2.95
85ARG	-2.75	341LEU	-2.80
466GLN	-2.73	80PHE	-2.50
80PHE	-2.55	338ARG	-2.47
83LEU	-2.23	46ARG	-2.31
348PHE	-2.04	85ARG	-2.16
90LEU	-2.03	79PHE	-2.14
230TYR	-1.99	61TRP	-2.02
79PHE	-1.80	65PRO	-1.96
643MET	-1.67	55TRP	-1.93
645LEU	-1.62	231ASN	-1.78
42ARG	-1.46	96PRO	-1.77
249LYS	-1.43	230TYR	-1.72
245VAL	-1.41	42ARG	-1.63
637LEU	-1.35	245VAL	-1.47
92ARG	-1.31	249LYS	-1.44
75ARG	-1.30	39ARG	-1.35
46ARG	-1.26	213ARG	-1.24
213ARG	-1.25	348PHE	-1.18
642GLU	-1.25	90LEU	-1.16
39ARG	-1.20	239THR	-1.10
239THR	-1.12	63PRO	-1.02

Table S4: Per-residue $\Delta\Delta G$ contributions for 8YF2 using AF3 ranking score vs. SAKE-PP.

**8YF2 – AF3 ranking score**		**8YF2 – SAKE-PP**	
**Residue**	$\Delta\Delta G$	**Residue**	$\Delta\Delta G$
155TYR	-5.25	83LYS	-7.05
171TYR	-3.85	17TYR	-4.44
69ARG	-3.15	12ARG	-3.38
152PHE	-2.79	121LEU	-3.38
121LEU	-2.76	171TYR	-3.36
83LYS	-2.36	87TYR	-3.28
74ARG	-2.35	138ILE	-3.20
122PHE	-2.24	120ARG	-2.96
164GLN	-2.12	155TYR	-2.81
110LYS	-2.09	175ARG	-2.71
120ARG	-2.03	123ARG	-2.60
123ARG	-1.91	156PHE	-2.52
167ASN	-1.89	122PHE	-2.47
115TYR	-1.68	124LYS	-2.46
124LYS	-1.48	69ARG	-2.41
153ASN	-1.23	132ARG	-2.18
		21ARG	-2.16
		110LYS	-1.99
		22LYS	-1.86
		117TYR	-1.63
		139TYR	-1.56
		74ARG	-1.55
		158LEU	-1.55
		166THR	-1.52
		75GLN	-1.42
		118LEU	-1.31

Table S5: Per-residue $\Delta\Delta G$ contributions for 8S4K using AF3 ranking score vs. SAKE-PP.

**8S4K – AF3 ranking score**		**8S4K – SAKE-PP**	
**Residue**	$\Delta\Delta G$	**Residue**	$\Delta\Delta G$
104TRP	-5.45	160PHE	-7.89
40TRP	-4.79	123TYR	-5.19
38LEU	-4.63	40TRP	-5.01
123TYR	-4.33	104TRP	-4.87
101PHE	-4.31	125LEU	-4.42
100HIE	-3.04	38LEU	-4.39
88PHE	-2.79	101PHE	-3.99
52TYR	-2.31	88PHE	-3.29
125LEU	-1.71	137LYS	-2.79
54ASN	-1.11	135LEU	-2.55
30VAL	-1.04	158HIE	-2.44
		52TYR	-2.33
		32GLN	-1.67
		131THR	-1.56
		163VAL	-1.20
		174SER	-1.15
		30VAL	-1.05
		165GLN	-1.01

Table S6: Per-residue $\Delta\Delta G$ contributions for 8SOZ using AF3 ranking score vs. SAKE-PP.

**8SOZ – AF3 ranking score**		**8SOZ – SAKE-PP**	
**Residue**	$\Delta\Delta G$	**Residue**	$\Delta\Delta G$
145ARG	-4.88	100ARG	-5.92
143TYR	-4.80	143TYR	-5.35
205TRP	-0.54	145ARG	-4.65
182LYS	-0.37	52TRP	-3.47
209TYR	-0.20	32TYR	-2.95
184TYR	-0.15	204PHE	-0.85
		53THR	-0.83
		205TRP	-0.71
		182LYS	-0.37
		209TYR	-0.27
		98GLN	-0.21
		50VAL	-0.16

Table S7: Per-residue $\Delta\Delta G$ contributions for 8XYZ using AF3 ranking score vs. SAKE-PP.

**8XYZ – AF3 ranking score**		**8XYZ – SAKE-PP**	
**Residue**	$\Delta\Delta G$	**Residue**	$\Delta\Delta G$
157TYR	-5.21	173TYR	-6.48
173TYR	-5.09	112LYS	-3.80
71ARG	-4.57	14ARG	-3.46
154PHE	-3.27	71ARG	-3.22
85LYS	-2.87	169ASN	-3.22
123LEU	-2.79	118ASN	-3.03
76ARG	-2.65	134ARG	-2.56
169ASN	-2.11	122ARG	-2.52
122ARG	-2.07	85LYS	-2.36
112LYS	-2.05	177ARG	-2.33
125ARG	-1.93	125ARG	-2.31
124PHE	-1.75	126LYS	-2.10
126LYS	-1.30	23ARG	-1.98
121TYR	-1.26	19TYR	-1.94
155ASN	-1.15	168THR	-1.92
		117TYR	-1.86
		158PHE	-1.77
		24LYS	-1.70
		76ARG	-1.69
		25ARG	-1.68
		140ILE	-1.56
		166GLN	-1.40
		138THR	-1.19
		157TYR	-1.14
		120LEU	-1.12
